# Structure variations within *R*Si_2_ and *R*_2_ *T*Si_3_ silicides. Part I. Structure overview

**DOI:** 10.1107/S2052520620001043

**Published:** 2020-03-12

**Authors:** M. Nentwich, M. Zschornak, M. Sonntag, R. Gumeniuk, S. Gemming, T. Leisegang, D. C. Meyer

**Affiliations:** a Institute for Experimental Physics, Technical University Bergakademie Freiberg, 09596 Freiberg, Germany; b Institute of Ion Beam Physics and Materials Research, Helmholtz-Zentrum Dresden-Rossendorf, 01328 Dresden, Germany; c Institute of Physics, Technische Universität Chemnitz, 09107 Chemnitz, Germany; d Samara Center for Theoretical Materials Science, Samara National Research University, 443086 Samara, Russia

**Keywords:** silicide, lanthanide, ordering phenomena, structure prediction, DFT

## Abstract

Here, structural parameters of various structure reports on *R*Si_2_ and *R*_2_ *T*Si_3_ compounds [where *R* is an alkaline earth metal, a rare earth metal (*i.e.* an element of the Sc group or a lathanide), or an actinide and *T* is a transition metal] are summarized. The parameters comprising composition, lattice parameters *a* and *c*, ratio *c*/*a*, formula unit per unit cell and structure type are tabulated. The relationships between the underlying structure types are presented within a group–subgroup scheme (Bärnighausen diagram). Additionally, unexpectedly missing compounds within the *R*_2_ *T*Si_3_ compounds were examined with density functional theory and compounds that are promising candidates for synthesis are listed. Furthermore, a correlation was detected between the orthorhombic AlB_2_-like lattices of, for example, Ca_2_AgSi_3_ and the divalence of *R* and the monovalence of *T*. Finally, a potential tetragonal structure with ordered Si/*T* sites is proposed.

## Abstract 2

Most articles dealing with *R*Si_2_ and *R*_2_ *T*Si_3_ compounds are only interested in one specific compound or in a series of compounds with varying *T* elements while keeping *R* fixed (or vice versa). Here, the focus lies on the complete space of 2:1:3 and 1:2 silicides. In addition further variations of superstructures are revealed and focus on crystallographic properties.

## 1. Introduction   

The rare earth disilicides *R*Si_2_ have been the subject of numerous studies in the past few decades mainly due to their exciting magnetic properties, such as magnetic ordering phenomena (Wang *et al.*, 2019 ▸; Pan *et al.*, 2013 ▸; Kotsanidis *et al.*, 1990 ▸; Li *et al.*, 1998*a* ▸, 2002*a* ▸, 2013 ▸; Bazela *et al.*, 2003 ▸; Inosov *et al.*, 2009 ▸), especially ferromagnetic ordering (Majumdar *et al.*, 1998 ▸, 1999*b* ▸; Li *et al.*, 1999 ▸, 2002*a* ▸,*b* ▸, 2003 ▸, 2013 ▸; Frontzek *et al.*, 2004 ▸), their spin-glass-like behavior (Li *et al.*, 1998*a* ▸, 1999 ▸, 2002*b* ▸, 2003 ▸; Kimura *et al.*, 1999 ▸; Szytuła *et al.*, 1999 ▸, 2000 ▸; Paulose *et al.*, 2003 ▸; Lu *et al.*, 2013 ▸) and Ruderman–Kittel–Kasuya–Yosida (RKKY) interactions (Li *et al.*, 2002*b* ▸; Inosov *et al.*, 2009 ▸; Tang *et al.*, 2010*a* ▸,*b* ▸; Lu *et al.*, 2013 ▸), which have been studied since the early 1980s. In the middle of the 20th century, ternary compounds of composition U_2_ *T*Si_3_ (with a transition metal *T* substituting one in four Si atoms) were a central research subject due to the emerging use of U-containing compounds in the military and the energy sector. Some of the formed structures are considered as prototypes for further *R*_2_ *T*Si_3_ compounds.

As it has been widely discussed in the literature (Hoffmann & Pöttgen, 2001 ▸; Pan *et al.*, 2013 ▸; Peter & Kanatzidis, 2012 ▸), the *R*Si_2_ and *R*_2_ *T*Si_3_ compounds crystallize with the hexagonal AlB_2_ and the tetragonal ThSi_2_ type and derivative structure types (Hoffmann & Pöttgen, 2001 ▸). Some of the disilicides are polymorphic (Perri *et al.*, 1959*b* ▸; Brown & Norreys, 1961 ▸; Mayer *et al.*, 1967 ▸), meaning that they crystallize in two or more different phases (International Union for Crystallography, 2017 ▸). This reflects in the now obsolete structure-type names α-USi_2_ and α-ThSi_2_ for tetragonal ThSi_2_ as well as β-USi_2_ and β-ThSi_2_ for hexagonal AlB_2_ (Evers *et al.*, 1980 ▸; Yashima *et al.*, 1982*a* ▸,*b* ▸,*c* ▸; Yashima & Satoh, 1982 ▸; Lejay *et al.*, 1983 ▸; Evers *et al.*, 1983 ▸; Weigel *et al.*, 1984 ▸; Sato *et al.*, 1984 ▸; Zhong *et al.*, 1985 ▸; Chevalier *et al.*, 1986 ▸; Dhar *et al.*, 1987 ▸).

The relationship between the large variety of the derivatives from AlB_2_ and ThSi_2_ aristotypes can be nicely explained within the group–subgroup scheme, also known as Bärnig­hausen formalism (Bärnighausen, 1980 ▸). The AlB_2_ structure is one of the simplest inorganic structure types. It has hexagonal space group *P*6/*mmm* (No. 191) and its unit cell incorporates only the two Wyckoff sites 1*a* and 2*d* (Hofmann & Jäniche, 1935 ▸) occupied by one *R* atom on the Al site and two Si atoms on the B site, forming a two-dimensional Si network, similar to graphite. The unit cell of the ThSi_2_ structure also has only two occupied Wyckoff positions (4*a* and 8*e*), but the Si sublattice forms a more complex 3D network (Brauer & Mittius, 1942 ▸).

Nowadays, 46 structure types derived from AlB_2_ (Hoffmann & Pöttgen, 2001 ▸) and four from ThSi_2_ are known. They include binary and ternary intermetallic compounds with compositions *RX*_2_, *RT*_2_, *RTX* or *R*_2_ *TX*_3_, where *X* is an element of the third or fourth group.

In this work, we systematize the occurrence of *R*Si_2_ and *R*_2_ *T*Si_3_ compounds, where *R* = alkaline earth metal, lanthanide, actinide or member of the Sc group and *T* is a transition metal. We present 12 different structure types of these compounds derived from the AlB_2_ type. Six of these structure types have not been considered by Hoffmann & Pöttgen (2001 ▸). Additionally, we present three further structure types based on the tetragonal ThSi_2_ type. One of these types is purely hypothetical and considers the possibility of ordered Si/*T* positions in ThSi_2_-like structures. Furthermore, we order all structure reports for *R*Si_2_ and *R*_2_ *T*Si_3_ compounds according to their *R* and *T* elements within an *R*–*T* grid. After analyzing all element combinations, we choose nine promising compounds not found in the literature and perform DFT calculations to evaluate the probability of a successful synthesis. We discuss peculiarities of the distribution of structure types among the *R*Si_2_ and *R*_2_ *T*Si_3_ compounds, based on a mapping of symmetries on the *R*–*T* grid with corresponding symbols.

## 2. Methods   

To gain a comprehensive overview of *R*Si_2_ and *R*_2_ *T*Si_3_ compounds, we performed an extensive literature search by scanning the ICSD, SciFinder and Reaxys databases for all possible element combinations for *T* within the Cr to Zn groups and *R* within the Sc group, the alkaline earth metal, the lanthanides and the actinides. Only experiments at ambient conditions were considered. Additionally, we did not consider data sets if they were too incomplete, *i.e.* missing lattice parameters or an insufficient description of the symmetry. Additionally, we did not take incommensurately modulated structures into account, because these modulations mainly arise for nonstoichiometric disilicides within this family of compounds and because the descriptions do not conform with those of conventional symmetry. Please refer to Leisegang (2010 ▸), Kubata *et al.* (2005 ▸) and Dshemuchadse (2008 ▸) for further information. However, commensurable modulations are interpreted as superstructures.

Table 1 ▸ contains the tabulated data of the composition of the compounds as well as their structure parameters, *i.e.* lattice parameters *a* and *c*, ratios *c*/*a*, formula units per unit cell, and structure type. These data were used without further refinement. The compounds, discussed within this article, are more than solid solutions as most of them exhibit ordered structures and, therefore, have distinct structure types compared to similar stoichiometries. Within this article, only the formula units and the deviation of the compounds within the range of *R* and *T* elements is of interest. Part II (Nentwich *et al.*, 2020 ▸) will discuss and compare other parameters.

We used calculations based on density functional theory (DFT) to predict the stability of not yet reported *R*Si_2_ and *R*_2_ *T*Si_3_ compounds. The formation energy Δ*E* ^tot^ is the difference of the total energy *E* ^tot^ of the compound and *E* ^tot^ of its elements, normalized to six atoms (*R*_2_Si_4_ or *R*_2_ *T*Si_3_). Appendix *B* [App appb] presents the space groups of the unary *R* crystals. The more negative the formation energy, the more thermodynamically favorable is the formation of that compound. We considered a formation energy of up to −25 meV per atom as potentially stable at room temperature. However, this assumption does not take into account potential energy barriers which might kinetically hinder the formation of the ground state. The projector-augmented wave (PAW) method (Kresse & Joubert, 1999 ▸) in spin-polarized Perdew–Burke–Ernzerhof parametrization (Perdew *et al.*, 1996 ▸) was employed as implemented in the *VASP* code (Kresse & Furthmüller, 1996 ▸). Total energies have been converged better than 10^−7^ eV with a maximum kinetic energy of 320 eV for the planewave basis set and Γ-centered *k*-point meshes with spacings less than 0.02 × 2π Å^−1^. All structures have been fully relaxed, with respect to atomic positions as well as cell geometry within the space group, to forces less than 10^−3^ V Å^−1^. A Hubbard *U* correlation correction was not used because the Si framework with *s*- and *p*-orbitals governs the stability of the structure and because it would complicate the comparability of the formation energies within the *R*_2_ *T*Si_3_ series.

## 3. Results and discussion   

In this article, we treat the *R*_2_ *T*Si_3_ compounds as a distinct phase with a fixed composition and not as a solid solution. As ternary phase diagrams are scarce for these compounds, we checked all available data, in particular the thermodynamic assessment of Bodak & Gladyshevskii (1985 ▸), for compositional degrees of freedom in the corresponding phase diagram region and possibly prevailing solid solutions. Nevertheless, the vast majority of compounds were reported to form superstructures which, in general, allow only slight variations in stoichiometry. We discuss those structures as distinct phases due to the changes in symmetry at these particular compositions in the phase diagrams. Many ternary phase diagrams are often determined at elevated temperatures, which is beyond the scope of this work. The phase diagrams given by Bodak & Gladyshevskii (1985 ▸) are not at room temperature.

### 3.1. Structural relationships   

**Figure 1:**
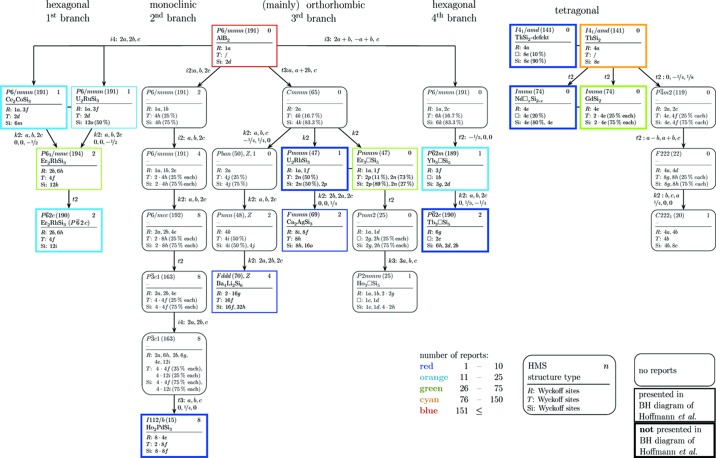
Bärnighausen diagram for *R*Si_2_ and *R*_2_ *T*Si_3_ compounds. The header of each box comprises the Hermann–Mauguin symbol of the space group, the range of ordering *n* and the structure type, whereas the body contains the occupied Wyckoff sites sorted by element. The arrows display the type of transformation between the structures: *t* is *translationengleich*, *k* is *klassengleich* and *i* is isomorphic. Fig. 2 ▸ comprises the respective structure plots. The fourth branch of AlB_2_-like compounds comprises the superstructures caused by interplays with vacancies (*R*_3_□Si_5_). A potential tetragonal superstructure is presented in the right-hand part of the diagram.

The many structure types within compounds *R*Si_2_ and *R*_2_ *T*Si_3_ compounds are related to each other according to their space groups and occupied Wyckoff positions. Starting from the highest symmetric structure, different perturbations induce symmetry reductions. Bärnighausen diagrams are the perfect tool to visualize these group–subgroup relationships in a simple and descriptive way. Fig. 1 ▸ presents the full Bärnighausen diagram for the *R*Si_2_ and *R*_2_ *T*Si_3_ compounds analyzed in this work. This diagram is partially based on a diagram by Hoffmann & Pöttgen (2001 ▸), but is greatly extended.

The presented Bärnighausen diagram would allow for further group–subgroup transitions; thus the authors cannot exclude the existence of further structure types within the *R*Si_2_ and *R*_2_ *T*Si_3_ compounds and thus also additional branches in the diagram. However, the space groups we present here already have a high number of free parameters. The extension of the diagram by further symmetry reduction accompanied with further degrees of freedom without losing the rough lattice and symmetry is challenging.

**Figure 2:**
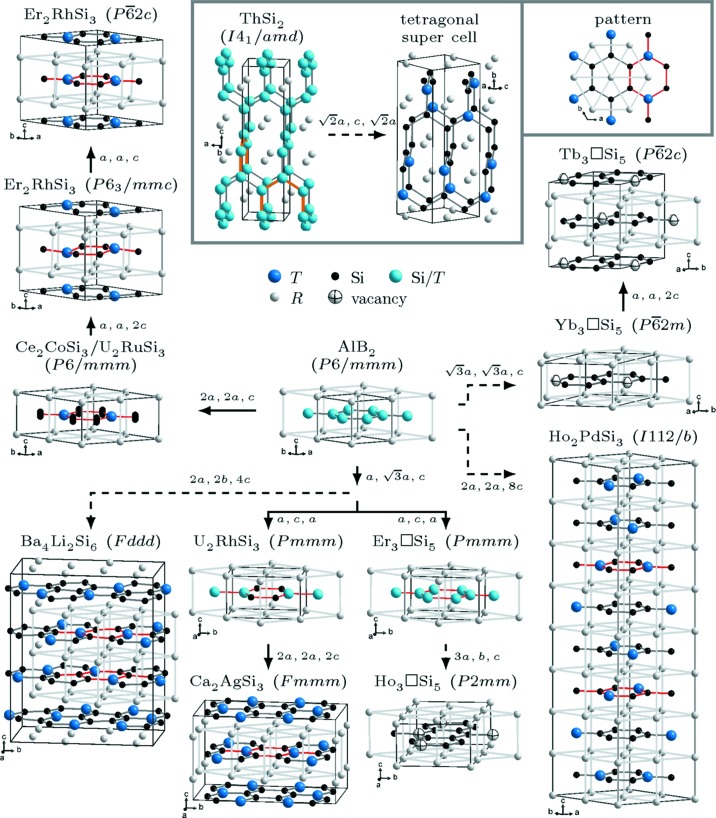
Models of the different observed structure types within *R*Si_2_ and *R*_2_ *T*Si_3_ compounds (unit cell outlined in black). The AlB_2_-like structures are depicted such that the view onto the two-dimensional *R* network is almost identical. The common structure pattern of the ordered AlB_2_-like structures (gray frame at right top) is highlighted with a light-gray frame and red Si/*T* bonds. The structure types Ce_2_CoSi_3_ and U_2_RuSi_3_ are almost identical. In contrast to the U_2_RuSi_3_ type, the Si atoms of the Ce_2_CoSi_3_ type are on the highly symmetric *z* = ½ position. This is highlighted by the blurred Si location along the *c* direction. The tetragonal structures (gray frame at center top) compose a 3D Si/*T* subnetwork with incomplete hexagons at the faces (highlighted in orange). The structures are connected according to their symmetry relations (dashed lines, if the transition is not minimal; labels comprise the lattice transformation).

Our diagram provides information about the type of transition (*klassengleiche* with perpetuation of lattice symmetry, *translationengleiche* with perpetuation of translational symmetry and isomorphous with perpetuation of both), the change of the lattice (direction and distance), the characteristics of the structure (space group, structure type and Wyckoff positions) as well as the absolute occurrence of the structure types in the literature. Additionally, Fig. 2 ▸ visualizes the atom arrangements of the different structures and presents their relationships in a hierarchical structure similar to the Bärnighausen diagram. In contrast, it focuses on the structural models and only shows these branches that include new structure types compared to Hoffmann & Pöttgen (2001 ▸). Appendix *A* [App appa] includes tables with Wyckoff positions of all structure types taken into account within this article (Tables 2 ▸, 3 ▸, 4 ▸, 5 ▸, 6 ▸, 7 ▸, 8 ▸, 9 ▸, 10 ▸, 11 ▸, 12 ▸, 13 ▸, 14 ▸, 15 ▸, 16 ▸ and 17 ▸).

#### 3.1.1. Compounds deduced from the AlB_2_ structure type   

First, we will present the relationships of *R*Si_2_ and *R*_2_ *T*Si_3_ compounds derived from the AlB_2_ structure. The lattice parameters are in the range of *a*_h_ ≈ 3.8–4.2 Å and *c*_h_ ≈ 3.9–4.5 Å, which is much higher than for the parent structure AlB_2_ itself (*a*_AlB_2__ = 3.00 Å, *c*_AlB_2__ = 3.24 Å).

Hoffmann & Pöttgen (2001 ▸) gave an overview of the hexagonal and orthorhombic transitions of AlB_2_-related compounds. Only three of Hoffmann’s Bärnighausen branches are applicable for the stoichiometries addressed here (*R*Si_2_ and *R*_2_ *T*Si_3_). We identify further structure types not discussed by Hoffmann & Pöttgen (2001 ▸), analyze the relationships of all structure types in the following paragraphs and show the new structure types in the Bärnighausen diagram (Fig. 2 ▸). Our Bärnighausen diagram (Fig. 2 ▸) thus exhibits four main branches which result from interactions with a *T* element or an Si vacancy □.

**The first branch** of the Bärnighausen diagram describes the symmetrical relationships between the hexagonal derivatives of the AlB_2_ type. Fig. 2 ▸ shows that Ce_2_CoSi_3_ (Gordon *et al.*, 1997 ▸) has the same structural motif as the aristotype. The difference is the ordering of the *T* atoms resulting in isolated [Si_6_] rings, see top right of Fig. 2 ▸. Only a certain part of this pattern is visible in the unit cell of Ce_2_CoSi_3_ and in other structure types of the *R*Si_2_ and *R*_2_ *T*Si_3_ compounds, indicated by red bonds. Besides [Si_6_] rings, [*T*_2_Si_4_] hexagons also occur, with the *T* atoms opposing each other in the ring. This ordering change indicates the doubling of the unit-cell parameter *a* in the Ce_2_CoSi_3_ type and an *isomorphous* symmetry reduction. If the Si atoms are shifted along the *c* direction, the layers are no longer perfectly planar, but puckered. This arrangement can be described with the same space group as Ce_2_CoSi_3_, but with half-occupied Wyckoff site 12*o*, instead of fully occupied 6*m*, known as the structure type U_2_RuSi_3_ (Pöttgen *et al.*, 1994 ▸). Fig. 2 ▸ shows both structure types within one subfigure with the different Si positions indicated by a series of atoms.

Compared to their ideal crystallographic positions, the Er_2_RhSi_3_ (*P*6_3_/*mmc*) type (Gladyshevskii *et al.*, 1992 ▸) exhibits shifts of the *T* atoms along the *c* direction accompanied by distortions of the *R* atoms centering the [*T*_2_Si_4_] rings. This puckering results in a doubling of the *c* parameter and thus a further *klassengleiche* reduction of the symmetry of the Ce_2_CoSi_3_ or U_2_RuSi_3_ type. The reported noncentrosymmetric structure for Er_2_RhSi_3_ (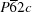) (Chevalier *et al.*, 1984 ▸) assumes additional distortions of the [Si_6_] rings and their centering *R* atoms by decoupled *x* and *y* coordinates resulting in a *translationengleiche* symmetry reduction of centrosymmetric Er_2_RhSi_3_ (*P*6_3_/*mmc*).

**The second branch** only includes the Ho_2_PdSi_3_ structure type (Tang *et al.*, 2011 ▸) with monoclinic space group *I*112/*b* (Nentwich *et al.*, 2016 ▸). This structure contains eight Si/*T* layers with stacking sequence *ABCDBADC*. Each layer exhibits the same Si/*T* occupation pattern as the Ce_2_CoSi_3_ type. The [*T*_2_Si_4_] rings of adjacent layers are shifted and rotated by multiples of 60° around the *c* axis with respect to each other. The 12-fold coordinated *R* elements are located on two different Wyckoff positions, either coordinated by two [*T*_2_Si_4_] rings or by one [*T*_2_Si_4_] ring and one [Si_6_] ring. The Ho_2_PdSi_3_ type contains 32 subcells and is thus one of the largest structures within the AlB_2_ Bärnighausen diagram. The atoms are assumed to be on the ideal crystallographic position, without any distortions, although the space group would allow this. The transition from AlB_2_ type to Ho_2_PdSi_3_ involves several symmetry reduction steps, detailed in Fig. 1 ▸.

**The third branch** comprises the orthorhombic derivatives of the AlB_2_ type. The starting point for further reductions is an orthohexagonal setting with space group *Cmmm* and Wyckoff sequence 2*a*, 4*k*. This setting is still a missing link (Hoffmann & Pöttgen, 2001 ▸), meaning that no report about a compound with this structure has been found. This space group has independent lattice parameters *a* and *b* – in contrast to all previous structure types – causing a *translationengleiche* symmetry reduction and making it an important starting point for five further structure types.

One of them is Ba_4_Li_2_Si_6_ (von Schnering *et al.*, 1996 ▸), which has perfectly ordered Si/*T* layers with the same occupational pattern as the Ce_2_CoSi_3_ type. As in the Ho_2_PdSi_3_ structure type, the Si/*T* atoms are perfectly ordered and form an *ABCD* stacking sequence, which is consistent with the two differently coordinated *R* sites as mentioned before. Accompanied with the anisotropic available space of the *R* site surrounded by one [*T*_2_Si_4_] and one [Si_6_] ring, its *z* component is not on the ideal crystallographic position resulting in a puckering of the *R* and Si/*T* layers. Identical *R* elements are connected along the former hexagonal *a* direction. These structural changes are accompanied with three consecutive *klassengleiche* symmetry reductions doubling the *a* and *b* parameters and quadrupling the *c* parameter.

A second structure type is U_2_RhSi_3_ (Pöttgen & Kaczorowski, 1993 ▸) with space group *Pmmm* (No. 47). Its Si/*T* atoms are partially ordered and only shifted along the *b* direction. These shifts induce a break in translational symmetry and a *klassengleiche* reduction. The Ho_2_PdSi_3_, Ba_4_Li_2_Si_6_ and Ca_2_AgSi_3_ structure types (Gordon *et al.*, 1997 ▸) have perfectly ordered Si/*T* layers and the same local arrangements around the *R* atoms. The *R* elements of the same Wyckoff site are connected along the orthorhombic *a* direction. These structural changes indicate the doubling of lattice parameters and a *klassengleiche* transition from structure type U_2_RhSi_3_. Hoffmann & Pöttgen (2001 ▸) have already reported a second structure type with the same space group as U_2_RhSi_3_, but with a different Wyckoff sequence, namely Er_3_□Si_5_. This type represents the disordered nonstoichiometric disilicides. In addition to the disordered ones, we also found reports about ordered versions. The otherwise very detailed review by Hoffmann & Pöttgen (2001 ▸) did not discuss these variants, which form due to vacancy ordering. According to the real stoichiometry of *R*Si_1.67_, one Si atom is regularly missing in the Si hexagons (Roge *et al.*, 1995 ▸). This arrangement can be realized by a hexagonal and a orthohexagonal setting (Auffret *et al.*, 1990 ▸). The hexagonal setting will be discussed in the fourth branch. The orthohexagonal arrangement requires a triplication of the *a* parameter. We will refer to this setting as Ho_3_□Si_5_ type. We prepared a list of its atomic parameters in space group *P*1 (No. 1) and inserted it to the software *FINDSYM* (Stokes & Hatch, 2005 ▸), which determined the highest possible space group as *Pmm*2 (No. 25). We changed the setting to *P*2*mm* (No. 25) for a better comparability to its supergroup *Pmmm* (No. 47). Thus, the triplication causes a *translationengleiche* and a *klassengleiche* symmetry reduction, which is accompanied with potential shifts of all atoms within the *a*,*b* plane.

**The fourth branch** comprises the ordered *R*_3_□Si_5_ structures, which are not related to the disordered Er_3_□Si_5_ type within the Bärnighausen diagram.

d’Avitaya *et al.* (1989 ▸) described a 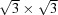 low-energy electron diffraction (LEED) pattern of Er_3_□Si_5_ thin films. Iandelli *et al.* (1979 ▸) determined the space group of this arrangement for Yb_3_□Si_5_ as 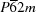 (No. 189), only allowing the *x* parameter of *R* and Si to deviate from its ideal crystallographic position. To consider the underlying symmetries of this arrangement, the cell needs to be enlarged and rotated with respect to the AlB_2_ unit cell using an *isomorphous* symmetry reduction. The location of the vacancy on an independent Wyckoff site is accompanied by a further *translationengleiche* symmetry reduction and an origin shift from space group *P*6/*mmm* to 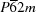.

Another model proposed by Stauffer *et al.* (1992 ▸) is based on the aforementioned arrangement, but every second Si/*T* layer is rotated by 120° around *c*. We determined the space group of this vacancy ordering as 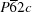, assuming that only the occupational pattern of the Si lattice would adapt, without changing the atomic positions. This results in a doubling of the *c* parameter, accompanied by a *klassengleiche* transition. The first reports concerning this arrangements used the compound Er_3_□Si_5_. However, this type name is already used for the disordered nonstoichiometric disilicides. Thus, we will refer to this structure type as Tb_3_□Si_5_ in accordance with the report by Luo *et al.* (1997 ▸).

We did not consider cells based on the Ho_3_□Si_5_ type with doubled *c* parameter, as it is only reported for the 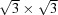 type cells.

**Further remarks**. Gordon *et al.* (1997 ▸) reported a further superstructure for Ce_2_PdSi_3_ with doubled lattice parameter *a* and quadrupled *c*, but did not focus on the specific space group. Therefore, we could not implement this report for the construction of the Bärnighausen diagram. During the literature research we additionally found structures of the EuGe_2_-type with space group 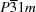 (No. 164). This structure type is very similar to the AlB_2_ type, but with a puckered Si sublattice, inducing a *translationengleiche* transition. Reports about this structure type refer to binary alkaline earth disilicides at non-ambient conditions (Evers *et al.*, 1977*b* ▸; Bordet *et al.*, 2000 ▸; Brutti *et al.*, 2006 ▸) or with mixed *R* sites (Eisenmann *et al.*, 1970 ▸; Evers *et al.*, 1979 ▸) as well as theoretical considerations about the puckering only (Gemming & Seifert, 2003 ▸; Gemming *et al.*, 2006 ▸; Enyashin & Gemming, 2007 ▸; Flores-Livas *et al.*, 2011 ▸). As these reports do not meet the requirements of experiments at ambient conditions, we did not consider this group of compounds within this work.

All aforementioned structure types will be termed AlB_2_-like in the following sections. By studying the atomic coordinates of the addressed space groups, we observed that the *R* elements form a rigid frame for the structure, as they are mostly the heaviest and largest elements in the structure and, thus, the most immobile. This also means that the Si/*T* atoms are more mobile and thus puckering of these layers is rather common.

#### 3.1.2. Compounds deduced from ThSi_2_ structure type   

Compounds of the ThSi_2_ type (Brauer & Mittius, 1942 ▸) crystallized in space group *I*4_1_/*amd* (No. 141), see gray box of Fig. 2 ▸ (with tetragonal lattice parameters *a*_t_ ≈ *a*_h_, *c*_t_ ≈ 13.4–14.4 Å). The Si/*T* atoms form a complex 3D network, in contrast to the 2D honeycombs in AlB_2_. So far, the only reported variation of the ThSi_2_ type is the GdSi_2_ structure (Perri *et al.*, 1959*b* ▸; Binder, 1960 ▸) with independent lattice parameters *a* and *b*. This degree of freedom causes a *translationengleiche* symmetry reduction to space group *Imma* (No. 74).

If the ThSi_2_ or GdSi_2_ type structures exhibit Si vacancies, these do not order regularly and only cause partially occupied Wyckoff positions. The proportion of vacancies is generally 10% (*R*Si_1.8_), thus almost one Si ion per tetragonal or orthorhombic unit cell is vacant. The resulting structures remain in the original space group and are called ThSi_2_-defect and Nd□_*x*_Si_2−*x*_, respectively.

In contrast to the distortive modulation of ThSi_2_, we did not find evidence for a tetragonal superstructure induced by ordering. This absence may be partially due to the small number of reports concerning tetragonal *R*_2_ *T*Si_3_ compounds [18 structure reports in ten articles (Gordon *et al.*, 1997 ▸; Albering *et al.*, 1994 ▸; Kaczorowski & Noël, 1993 ▸; Lejay *et al.*, 1983 ▸; Chevalier *et al.*, 1986 ▸; Li *et al.*, 2008 ▸; Mayer & Felner, 1973*b* ▸; Pöttgen & Kaczorowski, 1993 ▸; Raman & Steinfink, 1967 ▸; Raman, 1967 ▸)]. In order to shed light on a potential ordering, we constructed a tetragonal superstructure based on geometrical, chemical and electronic considerations. First, every Si atom has exactly one *T* element in its coordination. Second, every *T* element is coordinated by exactly three Si atoms. Third, every zigzag chain fulfills the 1:3 ratio of *T*:Si (zigzag chains explained in Section 3.2). And fourth, short-range periodicity is mandatory; thus, no doubling of the unit cell along the *c* direction is expected. By choosing an arbitrary atom within the tetragonal Si/*T* network as the first *T* element, only two positions unfold positioning the next *T* element. Two atomic arrangements resulted following the aforementioned conditions. We transferred these patterns onto the simple space group *P*1 (No. 1) and imported them into the tool *FINDSYM* (Stokes & Hatch, 2005 ▸) to determine the space group. Both variants proved to be identical and to exhibit the space group *C*222_1_ (No. 20). We will refer to this new structure type with eight instead of four formula units as POTS (proposed ordered, tetragonal structure). The gray box in Fig. 2 ▸ visualizes the Si/*T*-ordering. As this structure has not been reported so far for *R*_2_ *T*Si_3_ compounds, we decided to perform DFT calculations to estimate its stability, see Section 3.3.

These three structure types introduced in this section (§3.1.2) will be addressed as ThSi_2_-like in the following.

### 3.2. Structure description   

**Figure 3:**
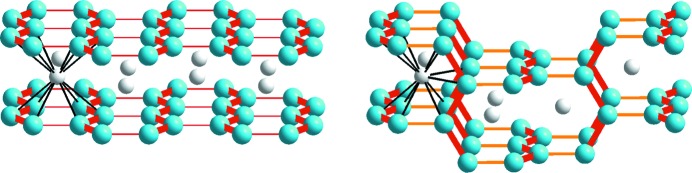
Differences in the arrangement of Si/*T* (blue) zigzag chains in hexagonal (left) and tetragonal (right) *R*Si_2_ and *R*_2_ *T*Si_3_ compounds. The consecutively added zigzag chains (red bonds) in hexagonal compounds always lie within the same plane, whereas in tetragonal compounds these layers are rotated by 90° along the bonds shown in orange. The 12-fold coordination of the *R* elements is highlighted for one atom ,as an example, with bonds shown in black.

The hexagonal and the tetragonal subgroups of *R*Si_2_ and *R*_2_ *T*Si_3_ compounds do not seem to be symmetrically related at first glance. The AlB_2_-like compounds exhibit graphite-like 2D networks of planar Si/*T* hexagons, whereas the Si/*T* atoms of ThSi_2_-like compounds form 3D networks. Still, the structures show similarities due to the trigonal coordination of the Si atoms. Fig. 3 ▸ illustrates the Si/*T* atoms in trigonal prisms, the 12-fold coordinated *R* atoms (connectors in black) and the Si/*T* zigzag chains (bonds in red/orange) in both structures.

Not only are the hexagonal honeycombs similar to graphite but also the tetragonal 3D network. The typical net exists simultaneously in planes perpendicular to the tetragonal *a*_t_ and *b*_t_ directions which are interconnected by bonds along the *c*_t_ direction. More precisely, two consecutive Si/*T* zigzag chains are rotated by 90° along the *c*_t_ direction, thereby spanning the (100)_t_ and (010)_t_ faces of the unit cell and causing incomplete hexagons (see the orange bonds in the ThSi_2_ structure type in Fig. 2 ▸). This additional symmetry degree of freedom causes a slight deformation of the trigonal Si/*T* arrangement in the tetragonal network. The Si—*T* bonds along the *c*_t_ direction (in orange, interchain) elongate in comparison to the intrachain bonds (in red), see Fig. 3 ▸. Further, the angle within the zigzag chains increases, whereas the other two angles decrease (between bonds shown in red and orange). Therefore, the chains with stronger bonds are slightly flattened compared to the ideal structure with perfect trigonal coordination. These structural differences between hexagonal and tetragonal structure types cause different crystal symmetries that permit a common origin in the Bärnighausen diagram for the *R*Si_2_ and *R*_2_ *T*Si_3_ compounds.

### 3.3. Elemental combinations and stability analysis of missing links with DFT calculations   

**Figure 4:**
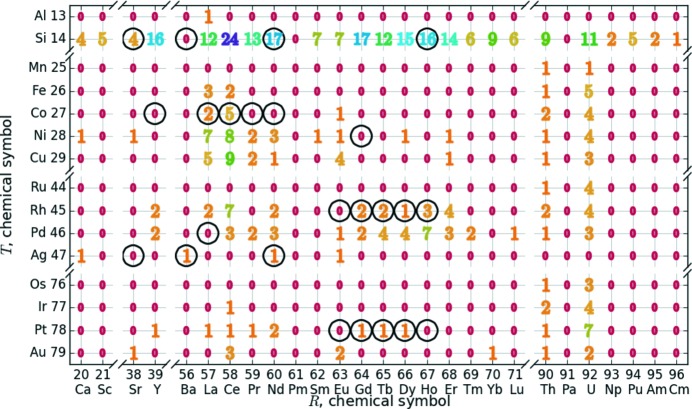
Overview of the literature reports of *R*Si_2_ and *R*_2_ *T*Si_3_ crystals. The number of reports is visualized with numbers and colors (few to very frequent: red – yellow – green – blue – purple). Additionally, to predict the stability for selected unreported structures, this study performed DFT calculations for the highlighted compounds (black circles).

During the literature search, we collected numerous structure reports of various *R*Si_2_ and *R*_2_ *T*Si_3_ compounds. Fig. 4 ▸ gives an overview of the reported compounds according to their appearance within the *R*–*T* grid. In this *R*–*T* diagram, we marked the number of reports with different colors, see Fig. 4 ▸. This diagram does not include the elements of the Zn group as those compounds were only analyzed at elevated temperatures (Demchenko *et al.*, 2002 ▸; Malik *et al.*, 2013 ▸; Nasir *et al.*, 2010 ▸; Romaka *et al.*, 2012 ▸; Salamakha *et al.*, 1998 ▸), which are out of the scope of this article. Additionally, we did not find any reports which include *R*_2_CrSi_3_ compounds. We assume that certain electron configurations are necessary for the formation of *R*_2_ *T*Si_3_ compounds. Furthermore, some elements rarely appear within the *R*_2_Si and *R*_2_ *T*Si_3_ compounds, such as Sm and Yb, which are highly volatile (Cao, 2014, private communication), Tc, which has a very low radio-active half-life and is very scarce (Holleman & Wiberg, 2007 ▸), or Pm, which is radioactive (Cao, 2014, private communication; Frontzek, 2014, private communication). The interest in using La and Lu was lower as most of the research aimed for the magnetic properties that do not exist for these two elements (Frontzek, 2014, private communication). The cost of the elements seems to play a subordinate role, *e.g.* the more expensive Rh (89 000 USD per kg) compounds were analyzed more frequently than the ones containing Ir (36 000 USD per kg) (Haynes, 2012 ▸).

**Figure 5:**
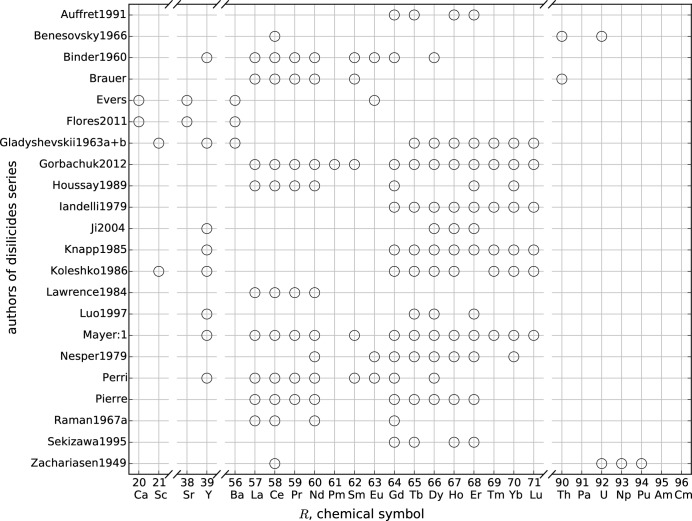
Overview of the *R*Si_2_ compounds that were analyzed systematically by the same first author. Some of the results were published in more than one article: Brauer (Brauer & Mittius, 1942 ▸; Brauer & Haag, 1950 ▸; Brauer & Haag, 1952 ▸), Evers (Evers *et al.*, 1977*a* ▸,*b* ▸, 1978*a* ▸,*b* ▸, 1983 ▸; Evers, 1979 ▸, 1980 ▸), Mayer:1 (Mayer *et al.*, 1962 ▸, 1967 ▸; Mayer & Eshdat, 1968 ▸), Perri (Perri *et al.*, 1959*a* ▸,*b* ▸), Pierre (Pierre *et al.*, 1988 ▸, 1990 ▸).

**Figure 6:**
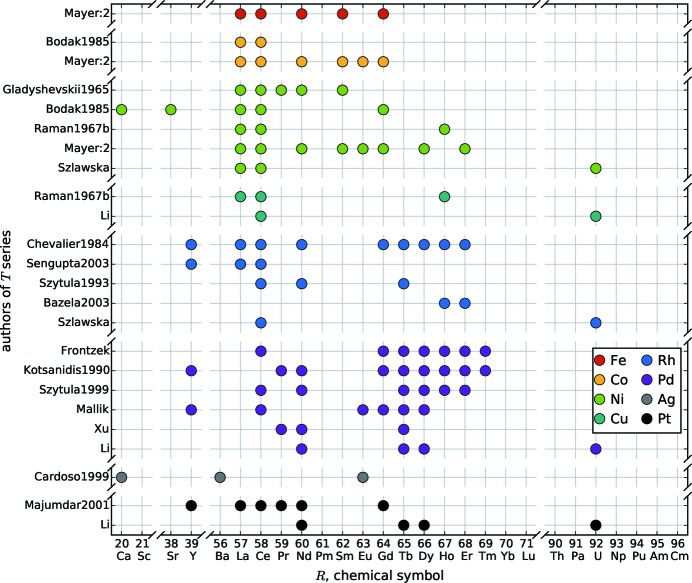
Overview of the *R*_2_ *T*Si_3_ compounds that were analyzed systematically by the same first author according to their *T* element (see color code). Some of the results were published in more than one article: Mayer:2 (Mayer & Tassa, 1969 ▸; Mayer & Felner, 1972 ▸, 1973*a* ▸,*b* ▸), Szlawska (Szlawska *et al.*, 2007 ▸, 2009 ▸, 2011 ▸, 2016 ▸; Szlawska & Kaczorowski, 2011 ▸, 2012 ▸), Li (Li *et al.*, 1997 ▸, 1998,*a* ▸,*b* ▸, 1999 ▸, 2001 ▸, 2002*a* ▸, 2003 ▸, 2008 ▸, 2013 ▸), Frontzek (Frontzek *et al.*, 2004 ▸, 2006 ▸; Frontzek, 2009 ▸), Mallik (Mallik & Sampathkumaran, 1996 ▸; Mallik *et al.*, 1998*a* ▸,*b* ▸,*c* ▸), Xu (Xu *et al.*, 2010 ▸, 2011*a* ▸,*b* ▸), Li (Li *et al.*, 1998*b* ▸, 2001 ▸, 2002*a* ▸, 2003 ▸, 2013 ▸).

**Figure 7:**
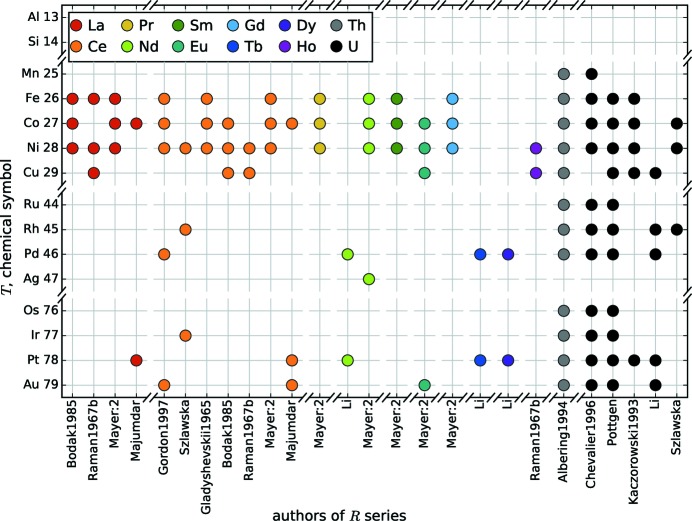
Overview of the *R*_2_ *T*Si_3_ compounds that were analyzed systematically by the same first author according to their *R* element (see color code). Some of the results were published in more than one article: Mayer:2 (Mayer & Tassa, 1969 ▸; Mayer & Felner, 1972 ▸, 1973*a* ▸,*b* ▸), Szlawska (Szlawska *et al.*, 2007 ▸, 2009 ▸, 2011 ▸, 2016 ▸; Szlawska & Kaczorowski, 2011 ▸, 2012 ▸), Li (Li *et al.*, 1997 ▸, 1998*a* ▸,*b* ▸, 1999 ▸, 2001 ▸, 2002*a* ▸, 2003 ▸, 2008 ▸, 2013 ▸), Frontzek (Frontzek *et al.*, 2004 ▸, 2006 ▸; Frontzek, 2009 ▸), Pottgen (Pöttgen & Kaczorowski, 1993 ▸; Pöttgen *et al.*, 1994 ▸), Majumdar (Majumdar *et al.*, 1998 ▸, 1999*a* ▸,*b* ▸, 2000 ▸, 2001 ▸).

These distributions are emphasized in Figs. 5 ▸, 6 ▸ and 7 ▸, which show systematic approaches in the literature. Fig. 5 ▸ gives an overview of *R*Si_2_ series with the corresponding authors and *R* elements. This summary shows the high interest in the lanthanide compounds compared to *R* elements of the alkaline earth metals and the actinides. Fig. 6 ▸ shows a similar illustration of *T* series within the *R*_2_ *T*Si_3_ compounds. Sorted by *T* element and author, the corresponding *R* elements are highlighted. Within the 3d elements the largest variety was analyzed, mostly in combination with La and Ce. In contrast, the heavy lanthanides were more favored when 4d elements were used, which have been intensively studied. Finally, Fig. 7 ▸ shows the *R* series, sorted by *R* element and author, with highlighted *T* elements. Again, the focus on the 3d elements as well as La and Ce is clear. The most complete investigations were carried out for U and Th, which emphasizes their importance for reactor technology.

By studying the *R*–*T* diagram of Fig. 4 ▸ one main question arises: What are the stability relationships of those *R*_2_ *T*Si_3_ compounds that are missing? To clarify this question, we sorted the compounds according their *R* element and discuss the Co, Rh and Pt series in the following sections.

We assumed ordered structures as DFT cannot evaluate mixed positions, except in the framework of virtual crystal approximations (VCA) using potential mixing. We adapted the structure type of the adjacent compounds within the *R*–*T* grid or used the highly symmetric Ce_2_CoSi_3_ structure type with space group *P*6/*mmm* (No. 191) as the basis for the unknown compounds. Table 17 ▸ summarizes the formation energies and lattice parameters all considered compounds. We will compare the formation energy of an unreported compound with those of similar reported compounds to evaluate its relative stability.

The DFT results of all models indicate metallic structures, although the DFT band gap problem may suppress the appearance of small band gaps. Thus, all structures have an intrinsic buffer of electronic states at the Fermi level to account for stability considerations of the *T* coordination within the ionic Si/*T* subnetwork according to molecular orbital theory, see Nentwich *et al.* (2020 ▸).

**Figure 8:**
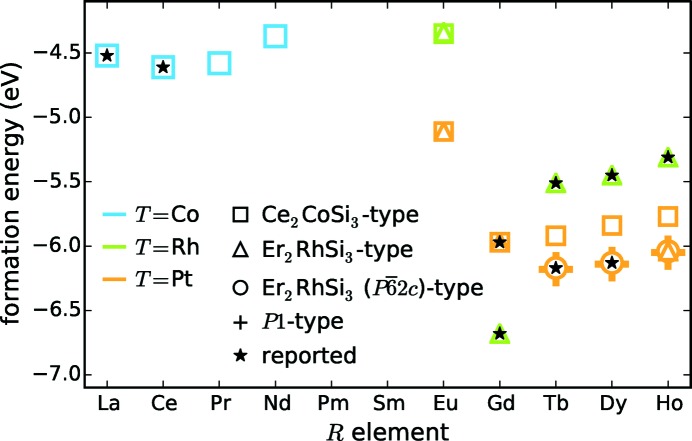
Formation energies of some *R*_2_ *T*Si_3_ compounds in different structure types.

The first compound of interest is Nd_2_CoSi_3_. The series of Nd compounds is fairly complete, compare Fig. 4 ▸, for example, with reported Nd_2_RhSi_3_ (Chevalier *et al.*, 1983 ▸, 1984 ▸; Szytuła *et al.*, 1993 ▸; Mitsufuji *et al.*, 1996 ▸; Gribanov *et al.*, 2010 ▸; Zajdel *et al.*, 2015 ▸), which is the 4d analog compound to Nd_2_CoSi_3_. Additionally, we found comments on this compound in two publications, but without any information concerning property, structure and phase purity (Chevalier *et al.*, 1984 ▸; Szytuła *et al.*, 1993 ▸). The formation energies and existing structure types of La_2_CoSi_3_ and Ce_2_CoSi_3_ serve as references. Furthermore, the likewise hypothetical compound Pr_2_CoSi_3_ was also calculated. The blue markers in Fig. 8 ▸ show the respective formation energies ranging from −4.61 eV to −4.37 eV. The lowest energy results for *R* = Ce and the highest for *R* = Nd. As the formation energy of Pr_2_CoSi_3_ lies in between the reported compounds, we expect it to be stable. The energy difference between Nd_2_CoSi_3_ and La_2_CoSi_3_ (the reported compound with highest energy) is 25 meV per atom. This corresponds to the tolerance limit; thus, we conclude that Nd_2_CoSi_3_ could also be stable. This conclusion is supported by the reports of Mayer & Tassa (1969 ▸) and Felner & Schieber (1973 ▸) on Pr_2_Co_0.8_Si_3.2_ and Nd_2_Co_0.8_Si_3.2_. They also synthesized samples with higher *T* content, which lead to ‘the disappearance of the AlB_2_ type phase, and the X-ray patterns obtained could not be interpreted’ (Mayer & Tassa, 1969 ▸). Nevertheless, we think that the synthesis of Pr_2_CoSi_3_ and Nd_2_CoSi_3_ and the interpretation of the corresponding X-ray patterns would be successful nowadays due to improved hardware and measurement techniques. Additionally, an enhanced thermal treatment would certainly improve the crystal quality regarding the Si/*T* ordering. Thus, we advise reinvestigating the *R*_2_ *T*Si_3_ compounds discussed by Mayer & Tassa (1969 ▸), with *R* = La, Ce, Pr, Nd, Sm, Gd and *T* = Fe, Co, Ni.

Another interesting compound is Eu_2_RhSi_3_. The Rh series is well represented in the *R*–*T* diagram and its 3d analog Eu_2_CoSi exists. However, the *R* element Eu supposedly only forms a compound with Co, but not with Rh (Mayer & Tassa, 1969 ▸; Mayer & Felner, 1973*a* ▸). We also modeled *R*_2_RhSi_3_ compounds with *R* elements Gd, Tb, Dy and Ho again and used the formation energies of existing structures as references. For the Rh series, the formation energies range from −6.68 eV to −4.34 eV, with the not yet reported Eu_2_RhSi_3_ having the highest formation energy. Both tested symmetries –the higher symmetric Ce_2_CoSi_3_ and the lower symmetric Er_2_RhSi_3_ – gave almost the same results, for formation energies (−4.34 eV) and interatomic distances [*d*_*a*_(*R*,*R*) ≈ 4.13 Å, *d*_*c*_(*R*,*R*) ≈ 4.27 Å]. The formation energy of Eu_2_RhSi_3_ differs from the second highest formation energy of Ho_2_RhSi_3_ by 160 meV per atom which exceeds the limit of 25 meV per atom, see green markers in Fig. 8 ▸. Therefore, the Eu_2_RhSi_3_ compound in Ce_2_CoSi_3_ or Er_2_RhSi_3_ structure type is significantly less stable.

The third compound of interest is Eu_2_PtSi_3_. In the *R*_2_PtSi_3_ series only a few element combinations have not yet been experimentally confirmed. Nevertheless, we identified missing compounds for *R* between Nd and Gd. Due to the radioactivity and low abundance of Pm and the volatility of Sm, we chose the Eu compound for further investigation. In analogy to the Rh series, we additionally chose *R* = Gd, Tb, Dy as references for formation energy and structure. In addition we modeled the not-yet-reported compound Ho_2_PtSi_3_. We decided to calculate the compounds in the reported Er_2_RhSi_3_ (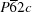) symmetry and additionally in the higher symmetric type Ce_2_CoSi_3_ as well as in the lowest possible symmetry *P*1 (No. 1) to evaluate the influence of the degrees of freedom onto the formation energies. The energies for the *R*_2_PtSi_3_ compounds range from −6.18 eV to −5.11 eV, see orange markers in Fig. 8 ▸. Except for Eu, the energies of different compounds and also different structure types are very similar. As expected, the energies of the lower symmetric Er_2_RhSi_3_ structure types are always lower than those of the highly symmetric type Ce_2_CoSi_3_, due to the additional degrees of freedom in atomic positions. The spread is between 0 meV for Gd and 28 meV for Ho per atom and about additional 1 meV going down to *P*1 (No. 1). The energies of the low-symmetric versions of the *R*_2_PtSi_3_ compounds are even lower than that of existing Gd_2_PtSi_3_. The formation energy of the (still) hypothetical Ho_2_PtSi_3_ in Ce_2_CoSi_3_ type structure is 33 meV per atom higher than that of Gd_2_PtSi_3_, thus this high-symmetry type is certainly not stable. However, the lower symmetry types will very probably be stable. The formation energy of Eu_2_PtSi_3_ is 14 meV per atom higher than for Gd_2_PtSi_3_; therefore, the compound is in the two considered symmetries most probably accessible as the thermodynamically stable phase. On the one hand, these data show that in some cases (Eu_2_RhSi_3_, Eu_2_PtSi_3_ and Gd_2_PtSi_3_) the formation energy hardly changes for different structure types. On the other hand, the formation energy of different structure types may change so strongly that our relative limit of 25 meV per atom is by far exceeded and only the lower symmetric variations may be stable. This is the case for Tb_2_PtSi_3_, Dy_2_PtSi_3_ and Ho_2_PtSi_3_.

After analyzing those three *R* series, we discovered further characteristics in the *R*–*T* diagram worth studying for different reasons. Compound La_2_PdSi_3_ attracted our attention because Chaika *et al.* (2001 ▸) and Behr *et al.* (2008 ▸) have already successfully synthesized this compound, but did not determine the lattice parameters or structural information during their investigations. We performed DFT calculations for La_2_PdSi_3_ using the Ce_2_CoSi_3_ structure type as well. The formation energy is lower than for the chemically similar compound La_2_CoSi_3_ which was reported in the ordered structure type Ce_2_CoSi_3_. Thus, we conclude that the Ce_2_CoSi_3_ type may be a stable configuration for La_2_PdSi_3_, next to the disordered AlB_2_ type. The relaxed parameters *a* = 8.34 Å and *c* = 4.38 Å are very close to the lengths expected from the adjacent compounds La_2_RhSi_3_ and Ce_2_PdSi_3_ (*a* ≈ 8.25 Å, *c* ≈ 4.3 Å). We recommend checking La_2_PdSi_3_ for indicators of an ordered Si/*T* site, *e.g.* satellite reflections.

Furthermore, we wondered which structure would arise for stoichiometric BaSi_2_. Most reported space groups of BaSi_2_ are orthorhombic (Imai & Watanabe, 2010 ▸; Evers, 1980 ▸; Janzon *et al.*, 1970 ▸; Kitano *et al.*, 2001 ▸; Migas *et al.*, 2007 ▸; Schäfer *et al.*, 1963 ▸; Evers *et al.*, 1977*b* ▸, 1978*a* ▸) and do not fit into our Bärnighausen diagram and are, therefore, not listed in Table 1 ▸ nor depicted in Figs. 4 ▸ and 9 ▸. The only exception is a hexagonal phase determined by Gladyshevskii (1959 ▸). In fact, the original sample had Li impurities and exhibits the structure type Ba_4_Li_2_Si_6_, discovered by von Schnering *et al.* (1996 ▸). This finding explains the discrepancy with the tetragonal phases of the related alkaline earth compounds CaSi_2_ and SrSi_2_, *e.g.* Evers *et al.* (1977*a* ▸,*b* ▸). We tested both an hexagonal and a tetragonal variant for BaSi_2_ to evaluate which symmetry is more stable. Additionally, we modeled SrSi_2_ in both the hypothetical AlB_2_ and the already reported ThSi_2_ structure type to compare the formation energies. As expected, the formation energy of tetragonal SrSi_2_ is lower than the one of hexagonal SrSi_2_. The energies for both BaSi_2_ models are almost identical (−2.06 eV) and, thus, expected to be equally stable. Nevertheless, these data alone are not sufficient to convey the stability of BaSi_2_ to SrSi_2_ as the elements Ba and Sr are too different. Furthermore, given the degrees of freedom, the tetragonal model of BaSi_2_ relaxed into an orthorhombic lattice with differences in lattice parameters *a* and *b* in the order of 0.4%. It should be noted that the *a* parameters of hexagonal and tetragonal symmetry differ for both BaSi_2_ and SrSi_2_ compounds (see Table 18 ▸), although they are alike for dimorphic compounds of the family, *e.g.* GdSi_2_.

Subsequently, we use the chemical similarity of Ba and Sr to evaluate which orthorhombic structure type is more favorable for compound Sr_2_AgSi_3_, as it is the only alkaline earth compound that has not yet been synthesized. Both, the Ba_4_Li_2_Si_6_ type of (Ba,Eu)_2_AgSi_3_ and the Ca_2_AgSi_3_ type are reasonable. We excluded other structure types as other chemically similar compounds only crystallize in those two structures. Here, chemically similar means a noble metal *T* and *R* preferring the +II oxidation state (*e.g.* alkaline earth metals, Eu and Yb). For *T* = Ag, Sr_2_AgSi_3_ is the only alkaline earth compound that has not yet been synthesized.

As a reference, we used Ba_2_AgSi_3_, also in both structure types. For Ba_2_AgSi_3_, the respective formation energies exhibited a clear preference for the reported Ca_2_AgSi_3_ type structure. However, the formation energies for both Sr_2_AgSi_3_ models are almost identical with a value of −2.83 eV, therefore we conclude that both structure types are equally stable. The formation energy of Sr_2_AgSi_3_ is slightly lower than that of Ba_2_AgSi_3_, which supports a stable structure.

Finally, we consider the potential tetragonal *R*_2_ *T*Si_3_ superstructure as determined in Section 3.1. We did not find reports on this ordered tetragonal structure and expect that it is energetically unfavored. Only a few articles on suitable compounds exist, mainly containing Th compounds (Albering *et al.*, 1994 ▸; Lejay *et al.*, 1983 ▸; Chevalier *et al.*, 1986 ▸; Li *et al.*, 2008 ▸; Raman, 1967 ▸; Kaczorowski & Noël, 1993 ▸; Pöttgen & Kaczorowski, 1993 ▸) as well as U_2_CuSi_3_ (Albering *et al.*, 1994 ▸; Lejay *et al.*, 1983 ▸; Chevalier *et al.*, 1986 ▸), La_2_AlSi_3_ (Raman & Steinfink, 1967 ▸), Ce_2_AuSi_3_ (Gordon *et al.*, 1997 ▸), Er_2_CuSi_3_ and Nd_2_AgSi_3_. We chose Nd_2_AgSi_3_ for better comparability, as several compounds with either Nd or Ag have already been examined in the previous discussions. To compare our hypothetical tetragonal superstructure with an existing structure, we chose the hexagonal Ce_2_CoSi_3_ type, since the most obvious tetragonal ThSi_2_ type exhibits mixed positions. We further took the disilicide NdSi_2_ into account in both ThSi_2_ and AlB_2_ type structures.

Please note that the lattice parameters of the POTS type (calculated) are related to those of the ThSi_2_ type (experimental) by rotation and elongation by a factor of 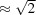. Thus, the interatomic distances of both tetragonal structure types of Nd_2_AgSi_3_ are approximately the same *a*_ThSi2_ = 4.12 Å ≈ 4.21 Å = *a*_POTS_/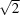. For Nd_2_CuSi_3_, we compared three different symmetries, the high symmetry Ce_2_CoSi_3_, experimentally confirmed Er_2_RhSi_3_ 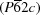 and low symmetry *P*1 (No. 1). The lattice parameters of all three models are *a* = 8.06 Å and * c* ≈ 4.24 Å, which is in good agreement with the experimental values [Er_2_RhSi_3_ 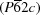-type].

The formation energies of Nd_2_AgSi_3_ stoichiometry are −3.69 eV for the Ce_2_CoSi_3_ type and −3.72 eV for the tetragonal superstructure. With an absolute formation energy which is lower by 0.30 eV per atom, the tetragonal type is clearly favored. In general, the superstructural order for tetragonal symmetries may be suppressed for further reasons. On the one hand, the 3D Si/*T* network itself may present kinetic barriers. On the other hand, the entropy of mixing may hinder structural ordering more severe for the degeneracies of the 3D Si/*T* network than for the planar stacking of hexagonal symmetries.

### 3.4. Structure distribution   

**Figure 9:**
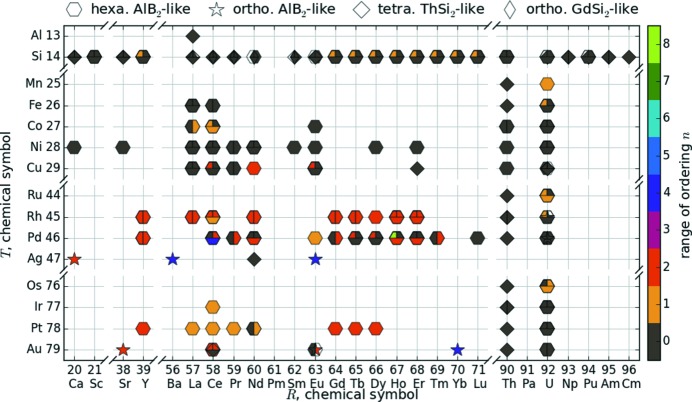
*R*–*T* diagram of the *R*Si_2_ and *R*_2_ *T*Si_3_ compounds. The color of the markers symbolizes the range of ordering *n*, see Section 3.4. If the structure is disordered (AlB_2_, ThSi_2_, GdSi_2_), then *n* = 0 and the symbol is gray. If the structure is ordered, the range of ordering accords to the number of stacks along *c* in the unit cell. Up to three markers on one grid position are possible, representing different publications.

Fig. 9 ▸ gives an overview of the scatter of structure types within the *R*Si_2_ and *R*_2_ *T*Si_3_ compounds. This figure adapts the *R*–*T* grid of Fig. 4 ▸ with symbols announcing symmetry and range of order. To quantify the ordering within the different structure types, we defined the range of order as zero if the Si/*T* atoms do not order and otherwise as the number of Si/*T* layers along *c* in the unit cell. The range of order is highlighted by the color of the marker. The symmetry is marked by shape: hexagon for hexagonal AlB_2_-like, open star for orthorhombic AlB_2_-like, diamond for tetragonal ThSi_2_, elongated diamond for orthorhombic GdSi_2_. For technical reasons, this diagram shows at most three reports of the same compound (left, right, bottom). Our algorithm chooses the datasets with the highest as well as the lowest *a* parameter and an additional dataset with a different structure type, to depict the most significant variations. Fig. 9 ▸ visualizes the range of order in dependence on the atomic number of the *R* and *T* cations; it depicts the following trends:

First, most of the compounds in the grid exhibit an hexagonal AlB_2_-like lattice. The other lattice types are mainly determined by the included *R* and *T* element. For example, the orthorhombic GdSi_2_ structure type arises exclusively for lanthanide disilicides. The tetragonal lattice is dominant for *R* = Th compounds as well as for the disilicides with light rare earth elements. Additional compounds with tetragonal lattice are Ce_2_AuSi_3_, Nd_2_AgSi_3_ and Er_2_CuSi_3_, all possessing a noble metal *T* element. Thus, the Fermi level of the *T* element affects the structural stability, see Nentwich *et al.* (2020 ▸).

Furthermore, the completely ordered orthorhombic structure types Ca_2_AgSi_3_ and Ba_4_Li_2_Si_6_ are only reported for *R*_2_ *T*Si_3_ compounds with the monovalent ions *T* = Ag, Au and the divalent ions *R* = Ca, Ba, Eu, Yb (Cardoso Gil *et al.*, 1999 ▸; Sarkar *et al.*, 2013 ▸). The partially ordered structure type U_2_RhSi_3_ additionally arises for U_2_PdSi_3_ (Chevalier *et al.*, 1996 ▸). Here, we do not consider the compound Ba_2_LiSi_3_ itself, since Li does not accord with our limitations to the *T* elements. Thus, the ordered orthorhombic AlB_2_-like structure types are more probable if the *T* element is a monovalent atom and if the *R* element prefers the +II oxidation state – as for the alkaline earth metals.

Second, tetragonal LaSi_2_ does not follow the hexagonal symmetry of the disilicides with third group elements Sc and Y. This phenomenon illustrates the affiliation of Sc and Y to the heavy and of La to the light rare earth elements (RÖMPP Online, 2011 ▸).

Third, with increasing atomic number of *R* within the lanthanide disilicides, three structure types succeed each other. The tetragonal ThSi_2_ type is the dominant one for light rare earth elements (Ce–Eu), followed by the orthorhombic GdSi_2_ type in the intermediate range and the hexagonal AlB_2_ type for the heavy rare earth elements (according to the classification by Sitzmann; RÖMPP Online, 2011 ▸). This development is present in all samples independent of their thermal treatment, see Nentwich *et al.* (2020 ▸). This meets an observation of Mayer *et al.* (1967 ▸): upon heating the samples to 1600°C, they discovered two phase transformations, one from AlB_2_ type to GdSi_2_ type and another one from GdSi_2_ type to ThSi_2_ type. These transformations are reversible. A decreasing atomic number within the lanthanide group is accompanied with a significantly increasing radius and therefore with a higher space requirement. Increased thermal lattice vibrations at higher temperatures also cause higher space requirements. Thus, annealing has the same effect as decreasing the atomic number of *R*.

## 4. Conclusions   

We present an extensive literature study of the *R*Si_2_ and *R*_2_ *T*Si_3_ compounds crystallizing in AlB_2_- and ThSi_2_-like structures complemented by DFT calculations. The local similarities between these structures, *e.g.* threefold planar coordination of the Si/*T* atoms, twelvefold coordination of the *R* elements, are highlighted and discussed. Additionally, we systematized the structure data and arranged them in a Bärnighausen diagram showing the relationships between structure types. We were able to determine the space groups of the ordered nonstoichiometric disilicides as piezoelectric 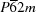 (No. 189), 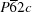 (No. 190) and *P*2*mm* (No. 25).

According to Bodak & Gladyshevskii (1985 ▸), compounds La_2_FeSi_3_, La_2_CoSi_3_, La_2_NiSi_3_, Ce_2_CuSi_3_ and Ce_2_NiSi_3_ form a solid solution of structure type AlB_2_ (disordered Si/*T* sites). Nevertheless, as evident from the discussion, we conclude that superstructures are expected to be the thermodynamic equilibrium structures, although they may be hard to synthesize, as they require obtaining the exact chemical composition on the one hand and for a careful thermal treatment on the other hand.

Comparison of the symmetry distribution within the *R*–*T* grid showed a special characteristic of the structure types Ca_2_AgSi_3_ and Ba_4_Li_2_Si_6_. These structure types only arise if *R* has the formal +II oxidation state and *T* is either Au or Ag. Additionally, these structures are reported to have ionic character, whereas all other compounds are reported to be metallic. The given *R*–*T* diagram also shows a transition from tetragonal ThSi_2_ to orthorhombic GdSi_2_ to hexagonal AlB_2_ type within the lanthanide disilicides with increasing atomic number of *R*. The structure types behave similarly with increasing temperature when respective crystals are heated.

Figs. 5 ▸ to 7 ▸ emphasize the number of systematic investigations of the *R*Si_2_ and *R*_2_ *T*Si_3_ compounds. On the one hand, these systematic investigations reduce systematic errors. On the other hand, the author’s expectations may also have an impact on the evaluation (such as the structure type).

Concluding the DFT analysis, hypothetical compounds Ho_2_PtSi_3_, Pr_2_CoSi_3_, Eu_2_PtSi_3_ and Nd_2_CoSi_3_ are suggested to be stable, whereas Eu_2_RhSi_3_ will be unstable. Due to the positive results for Pr_2_CoSi_3_ and Nd_2_CoSi_3_, we recommend reinvestigating the *R*_2_ *T*Si_3_ compounds reported by Mayer & Tassa (1969 ▸), with *R* = La, Ce, Pr, Nd, Sm, Gd and *T* = Fe, Co, Ni (originally with *R*_2_ *T*_0.8_Si_3.2_ stoichiometry). To complete the crystal structure information of La_2_PdSi_3_, we predict the lattice parameters *a* = 8.34 Å and *c* = 4.38 Å in a Ce_2_CoSi_3_ type structure. With respect to the question whether Sr_2_AgSi_3_ prefers the Ca_2_AgSi_3_ or the Ba_4_Li_2_Si_6_ structure type, both models result in almost identical formation energies of −2.83 eV and are equally stable from a theoretical point of view. Likewise, BaSi_2_ may exhibit hexagonal as well as tetragonal symmetry, as the formation energy of both models is −1.03 eV. In comparison, the potential tetragonal superstructure is less favorable than a highly symmetric hexagonal structure. The results of this work do not exclude the existence of structures that are equally or more stable than the ones presented here. The solid solutions with disorder at the Si/*T* position may always present potential candidates for the ground state of a specific *R*_2_ *T*Si_3_ compound.

At this point, the question of particular driving forces for a certain type of symmetry and the multiplicity of the superstructure symmetry types and structure types remains. This question will be addressed in the second part of this work (Nentwich *et al.*, 2020 ▸) focusing on the electronic structure.

## Figures and Tables

**Table 1:** Alphabetically sorted list of *R*Si_2_ and *R*_2_ *T*Si_3_ compounds and their crystal data *R* is an element of the alkaline earth metals, the scandium group, or the lanthanide or actinide series. *T* is a transition metal, Al or Si; thus a disilicide. The supercell can be identified by the formula units per unit cell. Lines written in blue indicate data sets not used for Fig. 9 ▸.

*R*	*T*	*a* (Å)	*b* (Å)	*c* (Å)	*c*/*a*	Formula units	Structure type	Thermal treatment	Reference	ICSD number
Am	Si	4.0190		13.6880	3.4058	4	ThSi_2_	–	Weigel *et al.* (1977 ▸)	
		4.0150		13.7330	3.4204	4	ThSi_2_	–	Weigel *et al.* (1984 ▸)	43816
Ba	Ag	8.6130	14.9270	19.6390	2.2802	16	Ba_4_Li_2_Si_6_	550°C, 1.5 days	Cardoso Gil *et al.* (1999 ▸)	410520
Ca	Ag	8.3150	8.6460	14.3910	1.7307	8	Ca_2_AgSi_3_	550°C, 1.5 days	Cardoso Gil *et al.* (1999 ▸)	410522
	Ni	3.9880		4.3460	1.0898	1	AlB_2_	–	Bodak & Gladyshevskii (1968 ▸)	20300
	Si	4.2830		13.5200	3.1567	4	ThSi_2_	–	Evers *et al.* (1977*a* ▸)	1453
		4.2830		13.5200	3.1567	4	ThSi_2_	–	Evers *et al.* (1978*b* ▸)	
		4.2832		13.5420	3.1617	4	ThSi_2_	–	McWhan *et al.* (1967 ▸)	87392
		4.2830		13.5300	3.1590	4	ThSi_2_	–	Nakano & Yamanaka (1994 ▸)	
Ce	Au	4.2220		14.3750	3.4048	4	*t*	750°C, 14 days	Gordon *et al.* (1997 ▸)	
		8.2840		8.7010	1.0503	8	*h*	750°C, 14 days	Gordon *et al.* (1997 ▸)	
		8.3060		8.6870	1.0459	8	Er_2_RhSi_3_ (190/194)	Floating zone	Majumdar *et al.* (2000 ▸)	
	Co	4.0440		4.1940	1.0371	1	AlB_2_	–	Bodak & Gladyshevskii (1985 ▸)	52846
		8.1040		4.1970	0.5179	4	Ce_2_CoSi_3_/U_2_RuSi_3_	750°C, 14 days	Gordon *et al.* (1997 ▸)	83895
		8.1100		4.2200	0.5203	4	Ce_2_CoSi_3_/U_2_RuSi_3_	750°C, 7 days	Majumdar *et al.* (1999*a* ▸)	
		8.1130		4.2190	0.5200	4	Ce_2_CoSi_3_/U_2_RuSi_3_	Floating zone	Majumdar *et al.* (2000 ▸)	
		8.0890		8.4020	1.0387	8	Er_2_RhSi_3_ (190/194)	800°C, 5 days	Patil *et al.* (2008 ▸)	
	Cu	4.0600		4.2800	1.0542	1	AlB_2_	–	Bodak & Gladyshevskii (1985 ▸)	
		4.0770		4.3140	1.0581	1	AlB_2_	–	Gladyshevskii & Bodak (1965 ▸)	20303
		4.0590		4.2940	1.0579	1	AlB_2_	–	Hwang *et al.* (1996 ▸)	
		4.0580		4.2960	1.0586	1	AlB_2_	850°C, 7 days	Lu *et al.* (2013 ▸)	
		8.0920		4.2060	0.5198	4	Ce_2_CoSi_3_/U_2_RuSi_3_	850°C, 7 days	Lu *et al.* (2013 ▸)	
		4.1360		4.2370	1.0244	1	AlB_2_	–	Raman (1967 ▸)	
		4.0650		4.3020	1.0583	1	AlB_2_	–	Raman (1967 ▸)	
		4.0640		4.3040	1.0591	1	AlB_2_	800°C, 7 days	Yubuta *et al.* (2009 ▸)	
		8.1280		8.6080	1.0591	8	Er_2_RhSi_3_ (190/194)	800°C, 7 days	Yubuta *et al.* (2009 ▸)	
	Fe	4.0680		4.1400	1.0177	1	AlB_2_	–	Gladyshevskii & Bodak (1965 ▸)	20304
		4.0620		4.2120	1.0369	1	*h*	750°C, 14 days	Gordon *et al.* (1997 ▸)	
	Ir	8.2120		4.2374	0.5160	4	Ce_2_CoSi_3_/U_2_RuSi_3_	–	Szlawska & Kaczorowski (2011 ▸)	
	Ni	4.0390		4.2870	1.0614	1	AlB_2_	–	Bodak & Gladyshevskii (1985 ▸)	621652
		4.0480		4.2910	1.0600	1	AlB_2_	–	Dhar *et al.* (1994 ▸)	658279
		4.0430		4.3020	1.0641	1	AlB_2_	–	Gladyshevskii & Bodak (1965 ▸)	20302
		4.0406		4.2801	1.0593	1	*h*	750°C, 14 days	Gordon *et al.* (1997 ▸)	
		4.0610		4.1490	1.0217	1	AlB_2_	–	Raman (1967 ▸)	
		4.0710		4.2020	1.0322	1	AlB_2_	–	Raman (1967 ▸)	
		4.0485		4.2887	1.0593	1	AlB_2_	800°C, 7 days	Rojas *et al.* 2010 ▸)	
		4.0450		4.2830	1.0588	1	AlB_2_	–	Szlawska & Kaczorowski (2012 ▸)	187100
	Pd	8.2631		17.1320	2.0733	16	*h*	750°C, 14 days	Gordon *et al.* (1997 ▸)	
		8.2330		8.5650	1.0403	8	Er_2_RhSi_3_ (190/194)	750°C, 7 days	Mallik & Sampathkumaran (1996 ▸)	
		4.1215		4.2723	1.0366	1	AlB_2_	750°C, 5 days	Szytuła *et al.* (1999 ▸)	
	Pt	8.2500		4.3320	0.5251	4	Ce_2_CoSi_3_/U_2_RuSi_3_	750°C, 14 days	Majumdar *et al.* (2001 ▸)	
	Rh	8.2100		8.4100	1.0244	8	Er_2_RhSi_3_	800°C, 4 days	Chevalier *et al.* (1984 ▸)	621958
		8.2310		8.4391	1.0253	8	Er_2_RhSi_3_	–	Kase *et al.* (2009 ▸)	
		8.3270		8.5160	1.0227	8	Er_2_RhSi_3_ ( 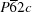 )	730°C, 4 days	Leciejewicz *et al.* (1995 ▸)	
		8.2370		8.4450	1.0253	8	Er_2_RhSi_3_ (190/194)	800°C, 5 days	Patil *et al.* (2008 ▸)	
		8.2300		8.4400	1.0255	8	Er_2_RhSi_3_ (190/194)	800°C, 5 days	Sengupta *et al.* (2003 ▸)	
		8.2240		4.2261	0.5139	4	Ce_2_CoSi_3_/U_2_RuSi_3_	–	Szlawska *et al.* (2009 ▸)	164827
		8.2620		8.4390	1.0214	8	Er_2_RhSi_3_ ( 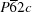 )	800°C, 54 days	Szytuła *et al.* (1993 ▸)	106425
	Si	4.1900		13.9300	3.3246	4	ThSi_2_	–	Benesovsky *et al.* (1966 ▸)	
		4.2700		13.8800	3.2506	4	ThSi_2_	–	Binder (1960 ▸)	
		4.1415		13.7816	3.3277	4	ThSi_2_	–	Brauer & Haag (1950 ▸)	622204
		4.1560		13.8400	3.3301	4	ThSi_2_	–	Brauer & Haag (1952 ▸)	25664
		4.1760		13.8480	3.3161	4	ThSi_2_-like	1100°C, 14 days	Dhar *et al.* (1987 ▸)	
		4.1910		13.8890	3.3140	4	ThSi_2_-defect	1100°C, 14 days	Dhar *et al.* (1987 ▸)	
		4.1940		13.9300	3.3214	4	ThSi_2_	–	Dijkman *et al.* (1982 ▸)	622206
		4.1900		13.9300	3.3246	4	ThSi_2_-defect or Nd□_*x*_Si_2−*x*_	800°C, 1 day	Houssay *et al.* (1989 ▸)	
		4.1900		13.8800	3.3126	4	ThSi_2_	–	Lahiouel *et al.* (1986 ▸)	622197
		4.2700		13.8800	3.2506	4	ThSi_2_	–	Lawrence *et al.* (1984 ▸)	622190
		4.1900		13.9400	3.3270	4	ThSi_2_	450°C, 0.5 days	Mayer *et al.* (1967 ▸)	622153
		4.1800		13.8900	3.3230	4	ThSi_2_	–	Mayer & Eshdat (1968 ▸)	
		4.1700		13.8200	3.3141	4	ThSi_2_-defect	950°C, 7 days	Murashita *et al.* (1991 ▸)	
		4.1900		13.9200	3.3222	4	ThSi_2_	950°C, 7 days	Murashita *et al.* (1991 ▸)	
		4.2700		13.8800	3.2506	4	*t*	–	Perri *et al.* (1959*b* ▸)	
		4.1900		13.9200	3.3222	4	ThSi_2_	–	Pierre *et al.* (1988 ▸)	
		4.1500		13.8700	3.3422	4	ThSi_2_	1000°C, 4 days	Raman & Steinfink (1967 ▸)	
		4.1920		13.9030	3.3166	4	ThSi_2_	–	Ruggiero & Olcese (1964 ▸)	622138
		4.1780		13.8500	3.3150	4	ThSi_2_-defect	1000°C, 3 days	Shaheen & Schilling (1987 ▸)	
		4.1880	4.1180	13.8800	3.3142	4	Nd□ _*x*_Si_2−*x*_	1000°C, 3 days	Shaheen & Schilling (1987 ▸)	622192
		4.1910		13.9490	3.3283	4	ThSi_2_	1000°C, 3 days	Shaheen & Schilling (1987 ▸)	622192
		4.1890		13.8920	3.3163	4	ThSi_2_	–	Weitzer *et al.* (1991 ▸)	622175
		4.1840		13.8560	3.3117	4	ThSi_2_	–	Yashima *et al.* (1982*c* ▸)	
		4.1600		13.9000	3.3413	4	ThSi_2_	–	Zachariasen (1949 ▸)	31642
Cm	Si	3.9630		13.7200	3.4620	4	ThSi_2_	–	Weigel & Marquart (1983 ▸)	
Dy	Ni	3.9700		4.0130	1.0108	1	AlB_2_	–	Mayer & Felner (1973*b* ▸)	53369
	Pd	8.1110		8.0550	0.9931	8	*h*	–	Kotsanidis *et al.* (1990 ▸)	
		4.0620		4.0310	0.9924	1	AlB_2_	750°C, 10 days	Li *et al.* (2003 ▸)	
		4.0620		4.0310	0.9924	1	AlB_2_	750°C, 10 days	Nimori & Li (2006 ▸)	
		4.0612		4.0334	0.9932	1	AlB_2_	750°C, 5 days	Szytuła *et al.* (1999 ▸)	
	Pt	8.1000		8.2000	1.0123	8	Er_2_RhSi_3_ ( 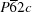 )	900°C, 23 days	Li *et al.* (2013 ▸)	
	Rh	8.0970		7.8230	0.9662	8	Er_2_RhSi_3_	800°C, 4 days	Chevalier *et al.* (1984 ▸)	630163
	Si	4.0400	3.9500	13.3300	3.2995	4	GdSi_2_	–	Binder (1960 ▸)	
		3.8300		4.1100	1.0731	1	AlB_2_	–	Gladyshevskii (1963 ▸)	20248
		3.8310		4.1210	1.0757	1	AlB_2_	700°C, 3 days	Iandelli *et al.* (1979 ▸)	630294
		3.8285	6.6312	4.1230	1.0769	2	Er_3_□Si_5_	1000°C, 10 days	Ji *et al.* (2004 ▸)	
		6.6338		4.1200	0.6211	3	Yb_3_□Si_5_	–	Knapp & Picraux (1985 ▸)	
		3.8310	6.6355	4.1210	1.0757	2	Er_3_□Si_5_	–	Koleshko *et al.* (1986 ▸)	53382
		3.8300		4.1200	1.0757	1	AlB_2_	450°C, 0.5 days	Mayer *et al.* (1967 ▸)	103369
		4.0450	3.9350	13.3190	3.2927	4	GdSi_2_	–	Mayer & Eshdat (1968 ▸)	630287
		4.0300	3.9300	13.3200	3.3052	4	GdSi_2_	–	Mayer & Eshdat (1968 ▸)	
		4.0300	3.9310	13.3200	3.3052	4	GdSi_2_	–	Mayer & Felner (1973*b* ▸)	
		3.9739		13.6760	3.4415	4	ThSi_2_	–	Nesper *et al.* (1979 ▸)	630314
		4.0400	3.9500	13.3400	3.3020	4	GdSi_2_	–	Perri *et al.* (1959*b* ▸)	630297
		4.0300		13.3800	3.3201	4	ThSi_2_	–	Perri *et al.* (1959*b* ▸)	150663
		4.0400	3.9500	13.3300	3.2995	4	GdSi_2_	–	Perri *et al.* (1959*a* ▸)	630297
		4.0380	3.9370	13.3100	3.2962	4	GdSi_2_	–	Pierre *et al.* (1988 ▸)	
Er	Cu	3.9670		13.7300	3.4611	4	ThSi_2_	–	Raman (1967 ▸)	627257
	Ni	3.9600		3.9860	1.0066	1	AlB_2_	–	Mayer & Felner (1973*b* ▸)	53404
	Pd	4.0640		3.9910	0.9820	1	*h*	Floating zone	Frontzek (2009 ▸)	
		8.0920		7.9250	0.9794	8	*h*	–	Kotsanidis *et al.* (1990 ▸)	
		4.0427		3.9794	0.9843	1	AlB_2_	750°C, 5 days	Szytuła *et al.* (1999 ▸)	
	Rh	8.0780		8.7480	1.0829	8	Er_2_RhSi_3_	800°C, 4 days	Bazela *et al.* (2003 ▸)	97376
		8.0780		7.7480	0.9591	8	Er_2_RhSi_3_ ( 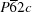 )	800°C, 4 days	Bazela *et al.* (2003 ▸)	97375
		8.0360		7.7120	0.9597	8	Er_2_RhSi_3_ ( 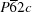 )	800°C, 4 days	Chevalier *et al.* (1984 ▸)	53413
		8.1130		7.7556	0.9559	8	Er_2_RhSi_3_	800°C, 14 days	Gladyshevskii *et al.* (1992 ▸)	300248
	Si	3.7930	6.5697	4.0820	1.0762	2	Er_3_□Si_5_	–	Auffret *et al.* (1990 ▸)	
		3.7990		4.0890	1.0763	1	AlB_2_	–	Gladyshevskii (1963 ▸)	20250
		3.7980		4.0880	1.0764	1	AlB_2_	700°C, 3 days	Iandelli *et al.* (1979 ▸)	631146
		3.7990	6.5801	4.0895	1.0765	2	Er_3_□Si_5_	1000°C, 10 days	Ji *et al.* (2004 ▸)	
		6.5818		4.0900	0.6214	3	Yb_3_□Si_5_	–	Knapp & Picraux (1985 ▸)	
		3.7990	6.5801	4.0900	1.0766	2	Er_3_□Si_5_	–	Koleshko *et al.* (1986 ▸)	631159
		3.7800		4.0900	1.0820	1	AlB_2_	–	Mayer *et al.* (1962 ▸)	631151
		3.7800		4.0800	1.0794	1	AlB_2_	450°C, 0.5 days	Mayer *et al.* (1967 ▸)	631140
		3.7850		4.0800	1.0779	1	AlB_2_	700°C, 2 days	Mayer & Felner (1972 ▸)	631144
		3.8000		4.0900	1.0763	1	AlB_2_	–	Mayer & Felner (1973*b* ▸)	631153
		3.9370		13.6160	3.4585	4	ThSi_2_	–	Nesper *et al.* (1979 ▸)	631164
		3.7920		4.0830	1.0767	1	AlB_2_	–	Pierre *et al.* (1988 ▸)	631150
		3.8000		4.0900	1.0763	1	AlB_2_	–	Sekizawa & Yasukouchi (1966 ▸)	631155
		6.5783		8.1760	1.2429	6	Tb_3_□Si_5_	700°C, 0 days	Tsai *et al.* (2005 ▸)	
Eu	Ag	8.4200	14.8580	17.8640	2.1216	16	Ba_4_Li_2_Si_6_	900°C, 3 days	Cardoso Gil *et al.* (1999 ▸)	410521
		4.1500		4.5150	1.0880	1	AlB_2_	–	Mayer & Felner (1973*a* ▸)	58453
		8.3060	9.0369	14.3770	1.7309	8	Ca_2_AgSi_3_	800°C, 5 days	Sarkar *et al.* (2013 ▸)	250524
	Co	4.0460		4.5000	1.1122	1	AlB_2_	–	Mayer & Felner (1973*a* ▸)	102379
	Cu	4.0762		4.4895	1.1014	1	AlB_2_-like	Floating zone	Cao *et al.* (2010 ▸, 2011 ▸)	
		8.1890		8.9760	1.0961	8	Er_2_RhSi_3_ (190/194)	800°C,	Majumdar *et al.* (1998 ▸)	
		4.0950		4.4880	1.0960	1	AlB_2_	–	Majumdar *et al.* (1999*b* ▸)	
		4.0800		4.4660	1.0946	1	AlB_2_	–	Mayer & Felner (1973*a* ▸)	53255
	Ni	4.0340		4.4960	1.1145	1	AlB_2_	–	Mayer & Felner (1973*a* ▸)	53436
	Pd	8.3188		4.3588	0.5240	4	Ce_2_CoSi_3_/U_2_RuSi_3_	750°C, 7 days	Rodewald *et al.* (2003 ▸)	391246
	Si	4.2900		13.3300	3.1072	4	ThSi_2_	–	Binder (1960 ▸)	631674
		4.3040		13.6500	3.1715	4	ThSi_2_	–	Evers *et al.* (1977*a* ▸)	1454
		4.3030		13.6600	3.1745	4	ThSi_2_	–	Evers *et al.* (1983 ▸)	
		4.0520		4.4820	1.1061	1	AlB_2_	–	Nesper *et al.* (1979 ▸)	103436
		4.2970		13.7040	3.1892	4	ThSi_2_	–	Nesper *et al.* (1979 ▸)	631683
		4.2900		13.6600	3.1841	4	*t*	–	Perri *et al.* (1959*b* ▸)	
Gd	Pd	4.0790		4.0980	1.0047	1	*h*	Floating zone	Frontzek (2009 ▸)	
		8.1580		8.1180	0.9951	8	*h*	750°C, 5 days	Kotsanidis *et al.* (1990 ▸)	
	Pt	8.1390		8.3030	1.0201	8	Er_2_RhSi_3_ (190/194)	750°C, 14 days	Majumdar *et al.* (2001 ▸)	
	Rh	8.1120		7.9760	0.9832	8	Er_2_RhSi_3_	800°C, 4 days	Chevalier *et al.* (1984 ▸)	636281
		8.1120		7.9760	0.9832	8	Er_2_RhSi_3_	–	Mulder *et al.* (1998 ▸)	
	Si	4.0920	4.0130	13.4370	3.2837	4	Nd□Si_2−*x*_	800°C, 1 day	Auffret *et al.* (1991 ▸)	
		4.0900	4.0100	13.4400	3.2861	4	GdSi_2_	–	Binder (1960 ▸)	
		4.0200	4.1000	13.4300	3.3408	4	Nd□_*x*_Si_2−*x*_	800°C, 1 day	Houssay *et al.* (1989 ▸)	636419
		3.8770		4.1720	1.0761	1	AlB_2_	700°C, 3 days	Iandelli *et al.* (1979 ▸)	636432
		6.7204		4.1700	0.6205	3	Yb_3_□Si_5_	–	Knapp & Picraux (1985 ▸)	
		3.8770	6.7152	4.1720	1.0761	2	Er_3_□Si_5_	–	Koleshko *et al.* (1986 ▸)	53633
		3.8700		4.1700	1.0775	1	AlB_2_	450°C, 0.5 days	Mayer *et al.* (1967 ▸)	636421
		4.0800	4.0100	13.4200	3.2892	4	GdSi_2_	–	Mayer & Eshdat (1968 ▸)	
		3.8690	6.7013	4.1820	1.0809	2	Er_3_□Si_5_	800°C, 14 days	Mulder *et al.* (1994 ▸)	658032
		3.8525		4.1470	1.0764	1	AlB_2_	–	Nesper *et al.* (1979 ▸)	636450
		4.0438		13.8020	3.4131	4	ThSi_2_	–	Nesper *et al.* (1979 ▸)	636452
		4.1000	4.0100	13.6100	3.3195	4	*o*	–	Perri *et al.* (1959*b* ▸)	150661
		4.0900	4.0100	13.4400	3.2861	4	GdSi_2_	–	Perri *et al.* (1959*a* ▸)	
		4.0900	4.0100	13.4400	3.2861	4	GdSi_2_	–	Pierre *et al.* (1988 ▸)	636434
		4.0930	4.0090	13.4400	3.2837	4	Nd□_*x*_Si_2−*x*_	–	Pierre *et al.* (1990 ▸)	
		4.0800	3.9960	13.4100	3.2868	4	GdSi_2_	1000°C, 4 days	Raman & Steinfink (1967 ▸)	
		4.0900	4.0100	13.4200	3.2812	4	GdSi_2_	–	Sekizawa & Yasukouchi (1966 ▸)	636440
Ho	Pd	8.1520		32.1680	3.9460	32	*h*	Floating zone	Frontzek (2009 ▸)	
		8.1010		7.9960	0.9870	8	*h*	750°C, 5 days	Kotsanidis *et al.* (1990 ▸)	
		8.0994		32.0192	3.9533	32	*h*	Floating zone	Leisegang (2010 ▸)	
		8.1072		8.1072	1.0000	8	Er_2_RhSi_3_ (190/194)	800°C, 7 days	Mo *et al.* (2015 ▸)	192586
		4.0459		3.9977	0.9881	1	AlB_2_	750°C, 5 days	Szytuła *et al.* (1999 ▸)	
		8.1000		32.0000	3.9506	32	Ho_2_PdSi_3_	Floating zone	Tang *et al.* (2011 ▸)	
		4.0460		3.9977	0.9881	4	Ce_2_CoSi_3_/U_2_RuSi_3_	750°C, 5 days	Zajdel *et al.* (2015 ▸)	
	Rh	8.0860		7.8040	0.9651	8	Er_2_RhSi_3_	800°C, 4 days	Bazela *et al.* (2003 ▸)	97374
		8.0860		7.8040	0.9651	8	Er_2_RhSi_3_ ( 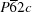 )	800°C, 4 days	Bazela *et al.* (2003 ▸)	97373
		8.0720		7.7710	0.9627	8	Er_2_RhSi_3_	800°C, 4 days	Chevalier *et al.* (1984 ▸)	639636
	Si	3.8070	6.5939	4.1060	1.0785	2	Er_3_□Si_5_	800°C, 1 day	Auffret *et al.* (1991 ▸)	
		4.0290	3.9170	13.2770	3.2954	4	Nd□_*x*_Si_2−*x*_	800°C, 1 day	Auffret *et al.* (1991 ▸)	
		3.8087	6.5969	4.1030	1.0773	2	Er_3_□Si_5_	1100°C, 8 days	Eremenko *et al.* (1995 ▸)	
		4.0230	3.9140	13.2820	3.3015	4	Nd□_*x*_Si_2−*x*_	1100°C, 8 days	Eremenko *et al.* (1995 ▸)	
		3.8160		4.1070	1.0763	1	AlB_2_	–	Gladyshevskii (1963 ▸)	20249
		3.8160		4.1070	1.0763	1	AlB_2_	700°C, 3 days	Iandelli *et al.* (1979 ▸)	639729
		3.8100	6.5991	4.1035	1.0770	2	Er_3_□Si_5_	1000°C, 10 days	Ji *et al.* (2004 ▸)	
		6.5991		4.1100	0.6228	3	Yb_3_□Si_5_	–	Knapp & Picraux (1985 ▸)	
		3.8160	6.6095	4.1070	1.0763	2	Er_3_□Si_5_	–	Koleshko *et al.* (1986 ▸)	639748
		4.0300	3.9700	13.3100	3.3027	4	*o*	–	Mayer *et al.* (1962 ▸)	
		3.8000		4.1000	1.0789	1	AlB_2_	450°C, 0.5 days	Mayer *et al.* (1967 ▸)	56250
		3.9610		13.6450	3.4448	4	ThSi_2_	–	Nesper *et al.* (1979 ▸)	639750
		4.0150	3.9060	13.2200	3.2927	4	GdSi_2_	–	Pierre *et al.* (1988 ▸)	639731
		3.9900	3.9400	13.3000	3.3333	4	GdSi_2_	–	Sekizawa & Yasukouchi (1966 ▸)	639743
		4.0280	3.9120	13.2870	3.2987	4	GdSi_2_	–	Weitzer *et al.* (1991 ▸)	
		4.0100	3.9120	13.2550	3.3055	4	GdSi_2_	–	Weitzer *et al.* (1991 ▸)	
La	Al	4.3030		14.2100	3.3023	4	ThSi_2_	1000°C, 4 days	Raman & Steinfink (1967 ▸)	
	Co	4.1880		4.3660	1.0425	1	AlB_2_	–	Bodak & Gladyshevskii (1985 ▸)	
		8.1850		4.3500	0.5315	4	Ce_2_CoSi_3_/U_2_RuSi_3_	750°C, 7 days	Majumdar *et al.* (1999*a* ▸)	
	Cu	4.0840		4.3950	1.0762	1	AlB_2_	–	Hwang *et al.* (1996 ▸)	
		4.1440		4.2860	1.0343	1	AlB_2_	–	Raman (1967 ▸)	103037
		4.0710		4.3830	1.0766	1	AlB_2_	–	Raman (1967 ▸)	
		4.0840		4.3950	1.0762	1	AlB_2_	–	Tien *et al.* (1997 ▸)	
	Fe	4.0800		4.3500	1.0662	1	AlB_2_	–	Bodak & Gladyshevskii (1985 ▸)	
		4.0690		4.1010	1.0079	1	AlB_2_	–	Raman (1967 ▸)	
		4.0970		4.3310	1.0571	1	AlB_2_	–	Raman (1967 ▸)	
	Ni	4.0930		4.3540	1.0638	1	AlB_2_	–	Bodak & Gladyshevskii (1985 ▸)	
		4.0770		4.3670	1.0711	1	AlB_2_	–	Gladyshevskii & Bodak (1965 ▸)	20305
		4.0450		4.3810	1.0831	1	AlB_2_	700°C, 2 days	Mayer & Felner (1972 ▸)	641574
		4.0770		4.3000	1.0547	1	AlB_2_	–	Raman (1967 ▸)	
		4.0570		4.3880	1.0816	1	AlB_2_	–	Raman (1967 ▸)	
		4.0711		4.3737	1.0743	1	AlB_2_	800°C, 7 days	Rojas *et al.* (2010 ▸)	
		4.0689		4.3753	1.0753	1	AlB_2_	–	Szlawska & Kaczorowski (2012 ▸)	
	Pt	8.2900		4.4170	0.5328	4	Ce_2_CoSi_3_/U_2_RuSi_3_	750°C, 14 days	Majumdar *et al.* (2001 ▸)	
	Rh	8.2330		8.5940	1.0438	8	Er_2_RhSi_3_	800°C, 4 days	Chevalier *et al.* (1984 ▸)	641751
		8.2800		8.6500	1.0447	8	Er_2_RhSi_3_ (190/194)	800°C, 5 days	Sengupta *et al.* (2003 ▸)	
	Si	4.3700		13.5600	3.1030	4	ThSi_2_	–	Bertaut & Blum (1950 ▸)	174010
		4.3100		13.2800	3.0812	4	ThSi_2_	–	Binder (1960 ▸)	
		4.2612		13.7118	3.2178	4	ThSi_2_	–	Brauer & Haag (1950 ▸)	641982
		4.2810		13.7500	3.2119	4	ThSi_2_	–	Brauer & Haag (1952 ▸)	25663
		4.3300		13.8300	3.1940	4	ThSi_2_-defect	800°C, 1 day	Houssay *et al.* (1989 ▸)	641955
		4.3100		13.8000	3.2019	4	ThSi_2_	–	Lawrence *et al.* (1984 ▸)	641973
		4.1900	4.2700	13.9400	3.3270	4	GdSi_2_	450°C, 0.5 days	Mayer *et al.* (1967 ▸)	641958
		4.2900		13.8700	3.2331	4	ThSi_2_	–	Mayer & Eshdat (1968 ▸)	641961
		4.3260		13.8400	3.1993	4	ThSi_2_	–	Nakano & Yamanaka (1994 ▸)	78028
		4.3100		13.8000	3.2019	4	*t*	–	Perri *et al.* (1959*b* ▸)	
		4.3000		13.8400	3.2186	4	ThSi_2_	–	Pierre *et al.* (1988 ▸)	
		4.3050		13.8400	3.2149	4	ThSi_2_	1000°C, 4 days	Raman & Steinfink (1967 ▸)	
Lu	Pd	4.0267		3.9218	0.9739	1	AlB_2_	Floating zone	Cao *et al.* (2013 ▸, 2014 ▸)	250596, 250597
	Si	3.7450		4.0500	1.0814	1	AlB_2_	–	Gladyshevskii (1963 ▸)	20253
		3.7470		4.0460	1.0798	1	AlB_2_	700°C, 3 days	Iandelli *et al.* (1979 ▸)	642610
		6.4952		4.0500	0.6235	3	Yb_3_□Si_5_	–	Knapp & Picraux (1985 ▸)	
		3.7450	6.4865	4.0500	1.0814	2	Er_3_□Si_5_	–	Koleshko *et al.* (1986 ▸)	642613
		3.7400		4.0400	1.0802	1	AlB_2_	–	Mayer *et al.* (1962 ▸)	642611
		3.7500		4.0500	1.0800	1	AlB_2_	450°C, 0.5 days	Mayer *et al.* (1967 ▸)	642607
Nd	Ag	4.1750		14.3100	3.4275	4	ThSi_2_	–	Mayer & Felner (1973*b* ▸)	605613
	Cu	8.0760		8.4400	1.0451	8	Er_2_RhSi_3_ (190/194)	800°C, 7 days	Yubuta *et al.* (2009 ▸)	
	Ni	4.0420		4.1630	1.0299	1	AlB_2_	–	Gladyshevskii & Bodak (1965 ▸)	20307
		4.0130		4.2020	1.0471	1	AlB_2_	700°C, 2 days	Mayer & Felner (1972 ▸)	76594
		4.0200		4.2070	1.0465	1	AlB_2_	–	Mayer & Felner (1973*b* ▸)	645635
	Pd	8.1970		8.4020	1.0250	8	*h*	750°C, 5 days	Kotsanidis *et al.* (1990 ▸)	
		4.1050		4.2040	1.0241	1	AlB_2_	750°C, 10 days	Li *et al.* (2003 ▸)	
		4.1033		4.2039	1.0245	1	AlB_2_	750°C, 5 days	Szytuła *et al.* (1999 ▸)	
	Pt	4.0927		4.2582	1.0404	1	AlB_2_	900°C, 23 days	Li *et al.* (2001 ▸)	
		8.2170		4.2820	0.5211	4	Ce_2_CoSi_3_/U_2_RuSi_3_	750°C, 14 days	Majumdar *et al.* (2001 ▸)	
	Rh	8.1860		8.2720	1.0105	8	Er_2_RhSi_3_	800°C, 4 days	Chevalier *et al.* (1984 ▸)	645781
		8.1710		8.2760	1.0129	8	Er_2_RhSi_3_ ( 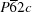 )	800°C, 54 days	Szytuła *et al.* (1993 ▸)	57432
	Si	4.1800	4.1500	13.5600	3.2440	4	GdSi_2_	–	Binder (1960 ▸)	
		4.1016		13.4223	3.2725	4	ThSi_2_	–	Brauer & Haag (1950 ▸)	645987
		4.1110		13.5600	3.2985	4	ThSi_2_	–	Brauer & Haag (1952 ▸)	25666
		4.1600	4.2000	13.6000	3.2692	4	Nd□_*x*_Si_2−*x*_	800°C, 1 day	Houssay *et al.* (1989 ▸)	645941
		4.1800	4.1500	13.5600	3.2440	4	GdSi_2_	–	Lawrence *et al.* (1984 ▸)	
		4.1700	4.1300	13.6500	3.2734	4	GdSi_2_	450°C, 0.5 days	Mayer *et al.* (1967 ▸)	645948
		4.1800	4.1600	13.6300	3.2608	4	GdSi_2_	–	Mayer & Eshdat (1968 ▸)	
		4.1800	4.1500	13.5600	3.2440	4	GdSi_2_	–	Mayer & Felner (1973*b* ▸)	
		4.1650		13.6420	3.2754	4	GdSi_2_	–	Nesper *et al.* (1979 ▸)	645989
		4.1110		13.5600	3.2985	4	ThSi_2_	–	Perri *et al.* (1959*b* ▸)	645972
		4.1740	4.1540	13.6100	3.2607	4	GdSi_2_	–	Pierre *et al.* (1988 ▸)	645963
		3.9480	6.8381	4.2690	1.0813	2	Er_3_□Si_5_	–	Pierre *et al.* (1990 ▸)	
		4.1350	4.1010	13.7400	3.3229	4	Nd□_*x*_Si_2−*x*_	–	Pierre *et al.* (1990 ▸)	
		4.1470	4.1250	13.6700	3.2964	4	Nd□_*x*_Si_2−*x*_	–	Pierre *et al.* (1990 ▸)	
		4.1620		13.5800	3.2629	4	ThSi_2_	1000°C, 4 days	Raman & Steinfink (1967 ▸)	645949
		4.1620		13.5800	3.2629	4	ThSi_2_	–	Raman (1968 ▸)	645985
		4.1850	4.1600	13.6100	3.2521	4	GdSi_2_	1050°C, 10 days	Schobinger-Papamantellos *et al.* (1991 ▸)	
Np	Si	3.9680		13.7150	3.4564	4	ThSi_2_	–	Yaar *et al.* (1992 ▸)	657647
		3.9700		13.7000	3.4509	4	ThSi_2_	–	Zachariasen (1949 ▸)	31644
Pr	Cu	4.0520		4.2550	1.0501	1	AlB_2_	–	Tien *et al.* (1997 ▸)	
		4.0420		4.2050	1.0403	1	AlB_2_	900°C, 20 days	Wang *et al.* (2014 ▸)	
	Ni	4.0450		4.2260	1.0447	1	AlB_2_	–	Gladyshevskii & Bodak (1965 ▸)	20306
		4.0210		4.0250	1.0010	1	AlB_2_	–	Mayer & Felner (1973*b* ▸)	646272
	Pd	8.2210		8.4660	1.0298	8	*h*	750°C, 5 days	Kotsanidis *et al.* (1990 ▸)	
		4.0250		4.2070	1.0452	1	AlB_2_	Floating zone	Xu *et al.* (2010 ▸)	
	Pt	8.2300		4.3000	0.5225	4	Ce_2_CoSi_3_/U_2_RuSi_3_	750°C, 14 days	Majumdar *et al.* (2001 ▸)	
	Si	4.2000		13.7600	3.2762	4	ThSi_2_	–	Binder (1960 ▸)	649371
		4.2100		13.7300	3.2613	4	ThSi_2_-defect	800°C, 5 days	Boutarek *et al.* (1994 ▸)	658012
		4.1315		13.4922	3.2657	4	ThSi_2_	–	Brauer & Haag (1950 ▸)	
		4.1480		13.6700	3.2956	4	ThSi_2_	–	Brauer & Haag (1952 ▸)	25665
		4.2100		13.7300	3.2613	4	ThSi_2_-defect	800°C, 1 day	Houssay *et al.* (1989 ▸)	649364
		4.2900		13.7600	3.2075	4	ThSi_2_	–	Lawrence *et al.* (1984 ▸)	
		4.1700	4.1200	13.8200	3.3141	4	GdSi_2_	450°C, 0.5 days	Mayer *et al.* (1967 ▸)	649365
		4.1600		13.7600	3.3077	4	ThSi_2_	–	Mayer & Eshdat (1968 ▸)	
		4.2900		13.7600	3.2075	4	ThSi_2_	–	Mayer & Felner (1973*b* ▸)	
		4.2000		13.7600	3.2762	4	ThSi_2_	–	Perri *et al.* (1959*b* ▸)	649376
		4.2000		13.7600	3.2762	4	ThSi_2_	–	Perri *et al.* (1959*a* ▸)	
		4.1840		13.7300	3.2815	4	ThSi_2_	–	Pierre *et al.* (1988 ▸)	
		4.2000		13.7300	3.2690	4	ThSi_2_-defect	–	Pierre *et al.* (1990 ▸)	
Pu	Si	3.9670		13.7200	3.4585	4	ThSi_2_	–	Coffinberry & Ellinger (1955 ▸)	649973
		3.8750	6.7117	4.1020	1.0586	2	Er_3_□Si_5_	840°C, 42 days	Land *et al.* (1965 ▸)	
		3.9680		13.7100	3.4551	4	ThSi_2_	–	Land *et al.* (1965 ▸)	649969
		3.8840		4.0820	1.0510	1	AlB_2_	–	Runnals & Boucher (1955 ▸)	44867
		3.9800		13.5800	3.4121	4	ThSi_2_	–	Zachariasen (1949 ▸)	31645
Sc	Si	3.6600	6.3393	3.8700	1.0574	2	Er_3_□Si_5_	–	Gladyshevskii & Émes-Misenko (1963 ▸)	
		3.6600	6.3393	3.8700	1.0574	2	Er_3_□Si_5_	–	Koleshko *et al.* (1986 ▸)	651822
		3.6620	6.3428	3.8790	1.0593	2	Er_3_□Si_5_	–	Kotroczo & McColm (1994 ▸)	
		3.6620	6.3428	3.8790	1.0593	2	Er_3_□Si_5_	–	Kotroczo & McColm (1994 ▸)	657975
		3.6600		3.8700	1.0574	1	AlB_2_	–	Nörenberg *et al.* (2006 ▸)	
Sm	Ni	4.0020		4.1600	1.0395	1	AlB_2_	–	Gladyshevskii & Bodak (1965 ▸)	20308
	Si	4.1050	4.0350	13.4600	3.2789	4	GdSi_2_	–	Binder (1960 ▸)	
		4.0417		13.3126	3.2938	4	ThSi_2_	–	Brauer & Haag (1950 ▸)	
		4.0490		13.3600	3.2996	4	ThSi_2_	–	Brauer & Haag (1952 ▸)	25667
		4.1100	4.0600	13.4900	3.2822	4	GdSi_2_	–	Mayer & Eshdat (1968 ▸)	652268
		4.1050	4.0350	13.4600	3.2789	4	*o*	–	Perri *et al.* (1959*b* ▸)	652273
		4.0800		13.5100	3.3113	4	ThSi_2_	–	Perri *et al.* (1959*b* ▸)	652274
		4.1040	4.0350	13.4600	3.2797	4	GdSi_2_	–	Perri *et al.* (1959*a* ▸)	
Sr	Au	8.3407	9.2664	14.4465	1.7320	8	Ca_2_AgSi_3_	650°C, 7 days	Zeiringer *et al.* (2015 ▸)	
	Ni	4.0690		4.6630	1.1460	1	AlB_2_	–	Bodak & Gladyshevskii (1968 ▸)	20301
	Si	4.4380		13.8300	3.1163	4	ThSi_2_	–	Evers *et al.* (1977*a* ▸)	1455
	Si	4.4380		13.8300	3.1163	4	ThSi_2_	–	Evers *et al.* (1978*b* ▸)	
		4.4290		13.8420	3.1253	4	ThSi_2_	–	Palenzona & Pani (2004 ▸)	99238
		4.4390		13.8380	3.1174	4	ThSi_2_	–	Palenzona & Pani (2004 ▸)	
Tb	Pd	4.0480		4.0370	0.9973	1	*h*	Floating zone	Frontzek (2009 ▸)	
		8.1210		8.1000	0.9974	8	*h*	750°C, 5 days	Kotsanidis *et al.* (1990 ▸)	
		4.0650		4.0520	0.9968	1	AlB_2_	750°C, 10 days	Li *et al.* (2003 ▸)	
		4.0643		4.0502	0.9965	1	AlB_2_	750°C, 5 days	Szytuła *et al.* (1999 ▸)	
	Pt	8.1223		8.2368	1.0141	8	Er_2_RhSi_3_ ( 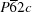 )	900°C, 23 days	Li *et al.* (2002*a* ▸)	
	Rh	8.1100		7.8600	0.9692	8	Er_2_RhSi_3_	800°C, 4 days	Chevalier *et al.* (1984 ▸)	650328
		8.1400		7.8120	0.9597	8	Er_2_RhSi_3_ ( 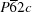 )	800°C, 54 days	Szytuła *et al.* (1993 ▸)	57483
	Si	3.8460	6.6615	4.1430	1.0772	2	Er_3_□Si_5_	800°C, 1 day	Auffret *et al.* (1991 ▸)	
		4.0570	3.9650	13.3770	3.2973	4	Nd□_*x*_Si_2−*x*_	800°C, 1 day	Auffret *et al.* (1991 ▸)	
		3.8470		4.1460	1.0777	1	AlB_2_	–	Gladyshevskii (1963 ▸)	20247
		3.8470		4.1460	1.0777	1	AlB_2_	700°C, 3 days	Iandelli *et al.* (1979 ▸)	652359
		6.6684		4.1500	0.6223	3	Yb_3_□Si_5_	–	Knapp & Picraux (1985 ▸)	
		3.8470	6.6632	4.1460	1.0777	2	Er_3_□Si_5_	–	Koleshko *et al.* (1986 ▸)	652375
		4.0450	3.9600	13.3800	3.3078	4	*o*	–	Mayer *et al.* (1962 ▸)	
		3.8400		4.1400	1.0781	1	AlB_2_	450°C, 0.5 days	Mayer *et al.* (1967 ▸)	652354
		3.9600	4.0500	13.3800	3.3788	4	GdSi_2_	–	Mayer & Eshdat (1968 ▸)	652355
		3.9902		13.6920	3.4314	4	ThSi_2_	–	Nesper *et al.* (1979 ▸)	652377
		4.0500	3.9650	13.3600	3.2988	4	GdSi_2_	–	Pierre *et al.* (1988 ▸)	652360
		4.0400	3.9600	13.3900	3.3144	4	GdSi_2_	–	Sekizawa & Yasukouchi (1966 ▸)	652370
Th	Au	4.1972		14.3030	3.4077	4	ThSi_2_	800°C, 7 days	Albering *et al.* (1994 ▸)	658096
	Co	4.0520		4.1510	1.0244	1	AlB_2_	800°C, 7 days	Albering *et al.* (1994 ▸)	658085
		4.0430		4.1890	1.0361	1	AlB_2_	950°C, 8 days	Zhong *et al.* (1985 ▸)	53078
	Cu	4.0230		4.1910	1.0418	1	AlB_2_	800°C, 7 days	Albering *et al.* (1994 ▸)	108410
	Fe	4.0993		14.1850	3.4603	4	ThSi_2_	800°C, 7 days	Albering *et al.* (1994 ▸)	658089
	Ir	4.1366		14.3640	3.4724	4	ThSi_2_	800°C, 7 days	Albering *et al.* (1994 ▸)	658094
		4.1200		14.3100	3.4733	4	ThSi_2_	–	Lejay *et al.* (1983 ▸), Chevalier *et al.* (1986 ▸)	
	Mn	4.1069		14.1130	3.4364	4	ThSi_2_	800°C, 7 days	Albering *et al.* (1994 ▸)	658088
	Ni	4.0322		4.1891	1.0389	1	AlB_2_	800°C, 7 days	Albering *et al.* (1994 ▸)	54299
	Os	4.1384		14.3784	3.4744	4	ThSi_2_	800°C, 7 days	Albering *et al.* (1994 ▸)	658093
	Pd	4.1570		14.2820	3.4357	4	ThSi_2_	800°C, 7 days	Albering *et al.* (1994 ▸)	658092
	Pt	4.1592		14.2850	3.4346	4	ThSi_2_	800°C, 7 days	Albering *et al.* (1994 ▸)	658095
	Rh	4.1241		14.3870	3.4885	4	ThSi_2_	800°C, 7 days	Albering *et al.* (1994 ▸)	658091
		4.1100		14.3200	3.4842	4	ThSi_2_	–	Lejay *et al.* (1983 ▸), Chevalier *et al.* (1986 ▸)	
	Ru	4.1242		14.4470	3.5030	4	ThSi_2_	800°C, 7 days	Albering *et al.* (1994 ▸)	658090
	Si	4.1180		14.2210	3.4534	4	ThSi_2_	–	Benesovsky *et al.* (1966 ▸)	
		4.1340		14.3750	3.4773	4	ThSi_2_	–	Brauer & Mittius (1942 ▸)	77320
		4.1260		14.3460	3.4770	4	ThSi_2_	–	Brauer & Mittius (1942 ▸)	660234
		4.1360		4.1260	0.9976	1	AlB_2_	–	Brown & Norreys (1959 ▸)	
		3.9850	6.9022	4.2280	1.0610	2	Er_3_□Si_5_	–	Brown & Norreys (1959 ▸)	
		4.1360		4.1260	0.9976	1	AlB_2_	–	Brown (1961 ▸)	15449
		4.1350		14.3750	3.4764	4	ThSi_2_	–	Brown (1961 ▸)	652390
		3.9850		4.2200	1.0590	1	AlB_2_	–	Jacobson *et al.* (1956 ▸)	26569
		4.1270		14.1940	3.4393	4	ThSi_2_	950°C, 8 days	Zhong *et al.* (1985 ▸)	
Tm	Pd	4.0570		3.9700	0.9786	1	*h*	Floating zone	Frontzek (2009 ▸)	
		8.0710		7.8500	0.9726	8	*h*	750°C, 5 days	Kotsanidis *et al.* (1990 ▸)	
	Si	3.7730		4.0700	1.0787	1	AlB_2_	–	Gladyshevskii (1963 ▸)	20251
		3.7680		4.0700	1.0801	1	AlB_2_	700°C, 3 days	Iandelli *et al.* (1979 ▸)	52468
		6.5298		4.0700	0.6233	3	Yb_3_□Si_5_	–	Knapp & Picraux (1985 ▸)	
		3.7730	6.5350	4.0700	1.0787	2	Er_3_□Si_5_	–	Koleshko *et al.* (1986 ▸)	604540
		3.7600		4.0700	1.0824	1	AlB_2_	–	Mayer *et al.* (1962 ▸)	652455
		3.7700		4.0700	1.0796	1	AlB_2_	450°C, 0.5 days	Mayer *et al.* (1967 ▸)	652451
U	Au	4.1450		3.9890	0.9624	1	AlB_2_	800°C, 60 days	Chevalier *et al.* (1996 ▸)	
		4.1450		3.9890	0.9624	1	AlB_2_	800°C, 8 days	Pöttgen & Kaczorowski (1993 ▸)	106295
	Co	3.9870		3.8830	0.9739	1	AlB_2_	800°C, 60 days	Chevalier *et al.* (1996 ▸)	
		3.9880		3.8830	0.9737	1	AlB_2_	800°C, 10 days	Kaczorowski & Noël (1993 ▸)	106494
		3.9880		3.8830	0.9737	1	AlB_2_	800°C, 8 days	Pöttgen & Kaczorowski (1993 ▸)	
		3.9765		3.8980	0.9803	1	AlB_2_	–	Szlawska *et al.* (2011 ▸)	
	Cu	3.9710		13.9260	3.5069	4	ThSi_2_	800°C, 10 days	Kaczorowski & Noël (1993 ▸)	603112
		4.0090		3.9570	0.9870	1	AlB_2_	600°C, 49 days	Pechev *et al.* (2000 ▸)	92357
		3.9710		13.9260	3.5069	4	ThSi_2_	800°C, 8 days	Pöttgen & Kaczorowski (1993 ▸)	602804
	Fe	4.0030		3.8570	0.9635	1	AlB_2_	800°C, 60 days	Chevalier *et al.* (1996 ▸)	
		4.0040		3.8640	0.9650	1	AlB_2_	800°C, 10 days	Kaczorowski & Noël (1993 ▸)	603109
		4.0100		3.8400	0.9576	1	AlB_2_	800°C, 7 days	Lourdes Pinto (1966 ▸)	53551
		4.0040		3.8640	0.9650	1	AlB_2_	800°C, 8 days	Pöttgen & Kaczorowski (1993 ▸)	
		8.0030		3.8540	0.4816	4	Ce_2_CoSi_3_/U_2_RuSi_3_	800°C, 10 days	Yamamura *et al.* (2006 ▸)	
	Ir	4.0650		3.9140	0.9629	1	AlB_2_	800°C, 60 days	Chevalier *et al.* (1996 ▸)	
		4.0720		3.8950	0.9565	1	AlB_2_	800°C, 8 days	Pöttgen & Kaczorowski (1993 ▸)	57398
		4.0830		3.9320	0.9630	1	AlB_2_-like	800°C, 7 days	Yubuta *et al.* (2006 ▸)	
		4.0900		3.8540	0.9423	1	AlB_2_-like	800°C, 7 days	Yubuta *et al.* (2006 ▸)	
	Mn	8.0450		3.8082	0.4734	4	Ce_2_CoSi_3_/U_2_RuSi_3_	800°C, 60 days	Chevalier *et al.* (1996 ▸)	
	Ni	3.9790		3.9460	0.9917	1	AlB_2_	800°C, 60 days	Chevalier *et al.* (1996 ▸)	
		3.9790		3.9490	0.9925	1	AlB_2_	800°C, 10 days	Kaczorowski & Noël (1993 ▸)	54300
		3.9790		3.9490	0.9925	1	AlB_2_	800°C, 8 days	Pöttgen & Kaczorowski (1993 ▸)	
		3.9720		3.9461	0.9935	1	AlB_2_	–	Schröder *et al.* (1995 ▸)	
	Os	8.1600		3.8440	0.4711	4	Ce_2_CoSi_3_/U_2_RuSi_3_	800°C, 60 days	Chevalier *et al.* (1996 ▸)	
		4.0666		3.8517	0.9472	1	AlB_2_	800°C, 8 days	Pöttgen & Kaczorowski (1993 ▸)	57453, 54310
		8.1600		3.8440	0.4711	4	Ce_2_CoSi_3_/U_2_RuSi_3_	800°C, 60 days	Pöttgen *et al.* (1994 ▸)	
	Pd	4.0800	7.0670	3.9390	0.9654	2	U_2_RhSi_3_	800°C, 60 days	Chevalier *et al.* (1996 ▸)	57172
		4.0830		3.9320	0.9630	1	AlB_2_	800°C, 3 days	Li *et al.* (1998*b* ▸)	
		4.0850		3.9350	0.9633	1	AlB_2_	800°C, 8 days	Pöttgen & Kaczorowski (1993 ▸)	57467
	Pt	4.0730		3.9650	0.9735	1	AlB_2_	800°C, 60 days	Chevalier *et al.* (1996 ▸)	
		4.0840		3.9730	0.9728	1	AlB_2_	800°C, 10 days	Kaczorowski & Noël (1993 ▸)	
		4.0810		3.9700	0.9728	1	AlB_2_	800°C, 10 days	Li *et al.* (1997 ▸)	
		4.0670		3.9640	0.9747	1	AlB_2_	800°C, 8 days	Pöttgen & Kaczorowski (1993 ▸)	602802
		4.0840		3.9730	0.9728	1	AlB_2_	850°C, 5 days	Sato *et al.* (1991 ▸)	54345
		4.0840		3.9730	0.9728	1	AlB_2_	–	Sato *et al.* (1992 ▸)	
		4.0730		3.9600	0.9723	1	AlB_2_	800°C, 10 days	Yamamura *et al.* (2006 ▸)	
	Rh	4.0620	7.0360	3.9290	0.9673	2	U_2_RhSi_3_	800°C, 60 days	Chevalier *et al.* (1996 ▸)	57171
		4.0740		3.8810	0.9526	1	AlB_2_	800°C, 3 days	Li *et al.* (1999 ▸)	
		4.0760		3.8830	0.9526	1	AlB_2_	800°C, 8 days	Pöttgen & Kaczorowski (1993 ▸)	57485
		8.1011		3.9477	0.4873	4	Ce_2_CoSi_3_	–	Szlawska *et al.* (2016 ▸)	
	Ru	8.1480		3.8550	0.4731	4	Ce_2_CoSi_3_/U_2_RuSi_3_	800°C, 60 days	Chevalier *et al.* (1996 ▸)	
		4.0750		3.8380	0.9418	1	AlB_2_	800°C, 8 days	Pöttgen & Kaczorowski (1993 ▸)	108727
		8.1450		3.8496	0.4726	4	Ce_2_CoSi_3_/U_2_RuSi_3_	800°C, 60 days	Pöttgen *et al.* (1994 ▸)	78530
		8.1480		3.8550	0.4731	4	Ce_2_CoSi_3_/U_2_RuSi_3_	800°C, 60 days	Pöttgen *et al.* (1994 ▸)	
	Si	3.8600		4.0700	1.0544	1	AlB_2_	–	Benesovsky *et al.* (1966 ▸)	
		3.9500		13.6800	3.4633	4	ThSi_2_	–	Benesovsky *et al.* (1966 ▸)	
		3.8520		4.0280	1.0457	1	AlB_2_	–	Brown & Norreys (1959 ▸)	652472, 52469
		3.8430	6.6563	4.0690	1.0588	2	Er_3_□Si_5_	–	Brown & Norreys (1959 ▸)	
		3.8520		4.0280	1.0457	1	AlB_2_	650°C	Brown & Norreys (1961 ▸)	
		3.8430	6.6563	4.0690	1.0588	2	Er_3_□Si_5_	650°C	Brown & Norreys (1961 ▸)	
		3.8390		4.0720	1.0607	1	AlB_2_	–	Dwight (1982 ▸)	106053
		3.8390		4.7200	1.2295	1	AlB_2_	–	Dwight (1982 ▸)	652476
		3.9220		14.1540	3.6089	4	ThSi_2_	–	Sasa & Uda (1976 ▸)	203
		3.8600		4.0700	1.0544	1	AlB_2_	–	Zachariasen (1949 ▸)	31646
		3.9800		13.7400	3.4523	4	ThSi_2_	–	Zachariasen (1949 ▸)	31643
Y	Pd	8.1380		8.0410	0.9881	8	*h*	750°C, 5 days	Kotsanidis *et al.* (1990 ▸)	
		8.0910		8.0920	1.0001	8	Er_2_RhSi_3_ (190/194)	750°C, 7 days	Mallik & Sampathkumaran (1996 ▸)	
	Pt	8.0990		8.1940	1.0117	8	Er_2_RhSi_3_ (190/194)	750°C, 14 days	Majumdar *et al.* (2001 ▸)	
	Rh	8.0860		7.8290	0.9682	8	Er_2_RhSi_3_	800°C, 4 days	Chevalier *et al.* (1984 ▸)	650353
		8.1300		7.8800	0.9692	8	Er_2_RhSi_3_ (190/194)	800°C, 5 days	Sengupta *et al.* (2003 ▸)	
	Si	3.8400	6.6511	4.1400	1.0781	2	Er_3_□Si_5_	–	Baptist *et al.* (1988 ▸)	
		6.6511		4.1400	0.6225	3	Yb_3_□Si_5_	–	Baptist *et al.* (1990 ▸)	
		4.0400	3.9500	13.3300	3.2995	4	GdSi_2_	–	Binder (1960 ▸)	
		3.8420	6.6545	4.1400	1.0776	2	Er_3_□Si_5_	–	Gladyshevskii & Émes-Misenko (1963 ▸)	
		3.8415	6.6537	4.1425	1.0784	2	Er_3_□Si_5_	1000°C, 10 days	Ji *et al.* (2004 ▸)	
		6.6511		4.1400	0.6225	3	Yb_3_□Si_5_	–	Knapp & Picraux (1985 ▸)	
		3.8420	6.6545	4.1400	1.0776	2	Er_3_□Si_5_	–	Koleshko *et al.* (1986 ▸)	652588
		3.8383		4.1310	1.0763	1	AlB_2_	–	Kotur & Mokra (1994 ▸)	658906
		4.0500	3.9500	13.2200	3.2642	4	GdSi_2_	–	Lazorenko *et al.* (1974 ▸)	652570
		3.8500		4.1400	1.0753	1	AlB_2_	–	Mayer *et al.* (1962 ▸)	652584
		4.0500	3.9500	13.4000	3.3086	4	*o*	–	Mayer *et al.* (1962 ▸)	
		3.8300		4.1400	1.0809	1	AlB_2_	450°C, 0.5 days	Mayer *et al.* (1967 ▸)	652566
		3.8430		4.1430	1.0781	1	AlB_2_	800°C, 2 days	Mayer & Felner (1972 ▸)	52478
		4.0400	3.9500	13.2300	3.2748	4	GdSi_2_	–	Perri *et al.* (1959*b* ▸)	652582
		4.0400		13.4200	3.3218	4	ThSi_2_	–	Perri *et al.* (1959*b* ▸)	150662
		4.0400	3.9500	13.3300	3.2995	4	GdSi_2_	–	Perri *et al.* (1959*a* ▸)	
Yb	Au	8.2003	14.1870	16.8690	2.0571	16	Ba_4_Li_2_Si_6_	800°C, 5 days	Sarkar *et al.* (2013 ▸)	250525
	Si	3.7710		4.0980	1.0867	1	AlB_2_	–	Gladyshevskii (1963 ▸)	20252
		3.7840		4.0980	1.0830	1	AlB_2_	700°C, 3 days	Iandelli *et al.* (1979 ▸)	52480
		6.5120		4.0900	0.6281	3	Yb_3_□Si_5_	700°C, 3 days	Iandelli *et al.* (1979 ▸)	
		6.5472		4.1000	0.6262	3	Yb_3_□Si_5_	–	Knapp & Picraux (1985 ▸)	
		3.7710	6.5316	4.0980	1.0867	2	Er_3_□Si_5_	–	Koleshko *et al.* (1986 ▸)	652601
		3.7700		4.1000	1.0875	1	AlB_2_	–	Mayer *et al.* (1962 ▸)	652598
		3.7610		4.0920	1.0880	1	AlB_2_	–	Nesper *et al.* (1979 ▸)	652603
		3.9868		13.5410	3.3965	4	ThSi_2_	850°C, 3 days	Peter & Kanatzidis (2012 ▸)	
		6.5120		4.0900	0.6281	3	Yb_3_□Si_5_	700°C, 21 days	Pöttgen *et al.* (1998 ▸)	

**Table 2:** Wyckoff positions of the hexagonal aristotypic structure type AlB_2_ with space group *P*6/*mmm* (No. 191) and lattice parameters *a*_h_ ≈ 3.00, *c*_h_ ≈ 3.24 Å

Element	Wyckoff symbol	*x*	*y*	*z*
*R*	1*a*	0	0	0
Si/*T*	2*d*	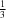	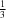	½

**Table 3:** Wyckoff positions of the hexagonal structure type Ce_2_CoSi_3_ with space group *P*6/*mmm* (No. 191) and lattice parameters *a* ≈ 2*a*_h_, *c* ≈ *c*_h_

Element	Wyckoff symbol	*x*	*y*	*z*
*R*	1*a*	0	0	0
*R*	3*f*	½	0	0
*T*	2*d*	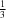	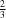	½
Si	6*m*	*x*_Si_ ≈ 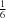	2*x*_Si_ ≈ 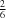	½

**Table 4:** Wyckoff positions of the hexagonal structure type U_2_RuSi_3_ with space group *P*6/*mmm* (No. 191) and lattice parameters *a* ≈ 2*a*_h_, *c* ≈ *c*_h_ The Si site is only half occupied.

Element	Wyckoff symbol	*x*	*y*	*z*
*R*	1*a*	0	0	0
*R*	3*f*	½	0	0
*T*	2*d*	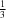	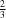	½
Si	12*o*	*x*_Si_ ≈ 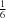	2*x*_Si_ ≈ 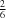	*z*_Si_ ≈ ½

**Table 5:** Wyckoff positions of the hexagonal structure type Er_2_RhSi_3_ with space group *P*6_3_/*mmc* (No. 194) and lattice parameters *a* ≈ 2*a*_h_, *c* ≈ 2*c*_h_

Element	Wyckoff symbol	*x*	*y*	*z*
*R*	2*b*	0	0	¼
*R*	6*h*	*x*_*R*_ ≈ ½	2*x*_*R*_ ≈ 0	¼
*T*	4*f*	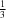	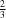	*z*_*T*_ ≈ 0
Si	12*k*	*x*_Si_ ≈ 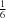	2*x*_Si_ ≈ 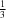	*z*_Si_ ≈ 0

**Table 6:** Wyckoff positions of the hexagonal structure type Er_2_RhSi_3_ with space group 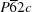 (No. 190) and lattice parameters *a* ≈ 2*a*_h_, *c* ≈ 2*c*_h_

Element	Wyckoff symbol	*x*	*y*	*z*
*R*	2*b*	0	0	¼
*R*	6*h*	*x*_*R*_ ≈ ½	*y*_*R*_ ≈ ½	¼
*T*	4*f*	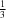	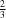	*z*_*T*_ ≈ 0
Si	12*h*	*x*_Si_ ≈ 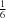	*y*_Si_ ≈ 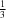	*z*_Si_ ≈ 0

**Table 7:** Wyckoff positions of the orthorhombic structure type Ho_2_PdSi_3_ with space group *I*112/*b* (No. 15) and lattice parameters *a* ≈ 2*a*_h_, *c* ≈ 8*c*_h_

Element	Wyckoff symbol	*x*	*y*	*z*
*R*	4*e*	0	¼	*z*_*R*,1_ ≈ 0
*R*	4*e*	0	¼	*z*_*R*,2_ ≈ 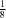
*R*	4*e*	0	¼	*z*_*R*,3_ ≈ 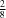
*R*	4*e*	0	¼	*z*_*R*,4_ ≈ 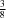
*R*	4*e*	0	¼	*z*_*R*,5_ ≈ 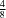
*R*	4*e*	0	¼	*z*_*R*,6_ ≈ 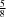
*R*	4*e*	0	¼	*z*_*R*,7_ ≈ 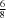
*R*	4*e*	0	¼	*z*_*R*,8_ ≈ 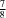
*T*	8*f*	*x*_*T*,1_ ≈ 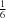	*y*_*T*,1_ ≈ 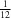	*z*_*T*,1_ ≈ 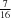
*T*	8*f*	*x*_*T*,2_ ≈ 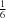	*y*_*T*,2_ ≈ 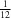	*z*_*T*,2_ ≈ 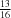
Si	8*f*	*x*_Si,1_ ≈ 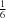	*y*_Si,1_ ≈ 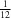	*z*_Si,1_ ≈ 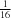
Si	8*f*	*x*_Si,2_ ≈ 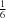	*y*_Si,2_ ≈ 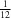	*z*_Si,2_ ≈ 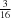
Si	8*f*	*x*_Si,3_ ≈ 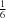	*y*_Si,3_ ≈ 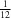	*z*_Si,3_ ≈ 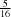
Si	8*f*	*x*_Si,4_ ≈ 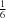	*y*_Si,4_ ≈ 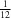	*z*_Si,4_ ≈ 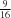
Si	8*f*	*x*_Si,5_ ≈ 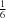	*y*_Si,5_ ≈ 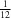	*z*_Si,5_ ≈ 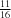
Si	8*f*	*x*_Si,6_ ≈ 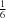	*y*_Si,6_ ≈ 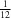	*z*_Si,6_ ≈ 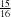

**Table 8:** Wyckoff positions of the orthorhombic structure type Er_3_□Si_5_ with space group *Pmmm* (No. 47) and lattice parameters *a* ≈ *a*_h_, *b* ≈ 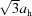, *c* ≈ *c*_h_

Element	Wyckoff symbol	*x*	*y*	*z*
*R*	1*a*	0	0	0
*R*	1*f*	½	½	0
Si/*T*	2*p*	½	*y*_Si/*T*,1_ ≈ ¼	½
Si/*T*	2*n*	0	*y*_Si/*T*,2_ ≈ ¼	½

**Table 9:** Wyckoff positions of the orthorhombic structure type U_2_RhSi_3_ with space group *Pmmm* (No. 47) and lattice parameters *a* ≈ *a*_h_, *b* ≈ 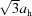, *c* ≈ *c*_h_

Element	Wyckoff symbol	*x*	*y*	*z*
*R*	1*a*	0	0	0
*R*	1*f*	½	½	0
Si/*T*	2*n*	0	*y*_*T*_ ≈ 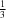	½
Si	2*p*	½	*y*_Si_ ≈ 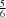	½

**Table 10:** Wyckoff positions of the orthorhombic structure type Ca_2_AgSi_3_ with space group *Fmmm* (No. 69) and lattice parameters *a* ≈ 2*a*_h_, *b* ≈ 2*c*_h_, *c* ≈ 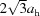

Element	Wyckoff symbol	*x*	*y*	*z*
*R*	8*i*	0	0	*z*_*R*_ ≈ ¼
*R*	8*f*	¼	¼	¼
*T*	8*h*	0	*y*_*T*_ ≈ 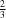	0
Si	8*h*	0	*y*_Si,1_ ≈ 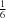	0
Si	16*o*	*x*_Si,2_ ≈ ¼	*y*_Si,2_ ≈ 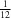	0

**Table 11:** Wyckoff positions of the orthorhombic structure type Ho_3_□Si_5_ with space group *P*2*mm* (No. 25) and lattice parameters *a* ≈ 3*a*_h_, 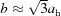, *c* ≈ 2*c*_h_

Element	Wyckoff symbol	*x*	*y*	*z*
*R*	1*a*	0	*y*_*R*,1_ ≈ 0	0
*R*	1*b*	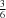	*y*_*R*,2_ ≈ ½	0
*R*	2*g*	*x*_*R*,1_ ≈ 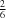	*y*_*R*,3_ ≈ 0	0
*R*	2*g*	*x*_*R*,2_ ≈ 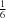	*y*_*R*,4_ ≈ ½	0
□	1*c*	0	*y*_□,1_ ≈ 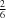	½
□	1*d*	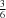	*y*_□,1_ ≈ 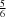	½
Si	1*c*	0	*y*_Si,1_ ≈ 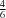	½
Si	1*d*	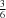	*y*_Si,2_ ≈ 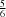	½
Si	2*h*	*x*_Si,1_ ≈ 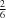	*y*_Si,3_ ≈ 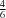	½
Si	2*h*	*x*_Si,2_ ≈ 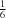	*y*_Si,4_ ≈ 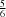	½
Si	2*h*	*x*_Si,3_ ≈ 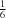	*y*_Si,5_ ≈ 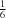	½
Si	2*h*	*x*_Si,4_ ≈ 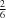	*y*_Si,6_ ≈ 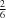	½

**Table 12:** Wyckoff positions of the orthorhombic structure type Ba_4_Li_2_Si_6_ with space group *Fddd* (No. 70) and lattice parameters *a* ≈ 2*a*_h_, 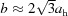, *c* ≈ 4*c*_h_

Element	Wyckoff symbol	*x*	*y*	*z*
*R*	16*g*	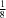	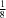	*z*_*R*,1_ ≈ 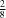
*R*	16*g*	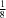	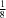	*z*_*R*,2_ ≈ 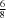
*T*	16*f*	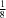	*y*_*T*_ ≈ 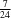	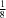
Si	16*f*	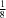	*y*_Si,1_ ≈ 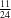	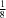
Si	32*h*	*x*_Si,2_ ≈ 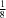	*y*_Si,2_ ≈ 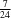	*z*_Si,2_ ≈ 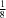

**Table 13:** Wyckoff positions of the Si vacancy cell of structure type Yb_3_□Si_5_ with space group 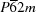 (No. 189) and lattice parameters 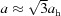, *c* ≈ *c*_h_

Element	Wyckoff symbol	*x*	*y*	*z*
*R*	3*f*	*x*_*R*_ ≈ 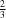	0	0
□	1*b*	0	0	½
Si	3*g*	*x*_Si_ ≈ 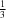	0	½
Si	2*d*	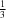	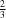	½

**Table 14:** Wyckoff positions of the Si vacancy cell of structure type Tb_3_□Si_5_ with space group 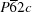 (No. 190) and lattice parameters 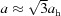, *c* ≈ 2*c*_h_

Element	Wyckoff symbol	*x*	*y*	*z*
*R*	6*g*	*x*_*R*_ ≈ 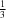	0	0
□	2*c*	0	0	¼
Si	6*h*	*x*_Si_ ≈ 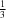	*y*_Si_ ≈ 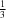	¼
Si	2*d*	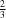	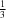	¼
Si	2*b*	0	0	¼

**Table 15:** Wyckoff positions of the tetragonal structure type ThSi_2_ with space group *I*4_1_/*amd* (No. 141) with lattice parameters *a*_t_ ≈ *a*_h_, *c*_t_ ≈ 13.4–14.4 Å

Element	Wyckoff symbol	*x*	*y*	*z*
*R*	4*a*	0	0	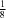
Si/*T*	8*e*	0	0	*z*_Si/*T*_ ≈ 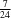

**Table 16:** Wyckoff positions of the orthorhombic structure type GdSi_2_ with space group *Imma* (No. 74) and lattice parameters *a* ≈ *a*_t_, *c* ≈ *c*_t_

Element	Wyckoff symbol	*x*	*y*	*z*
*R*	4*e*	0	¼	*z*_*R*_ ≈ 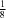
Si/*T*	4*e*	0	¼	*z*_Si/T,1_ ≈ 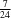
Si/*T*	4*e*	0	¼	*z*_Si/T,2_ ≈ 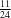

**Table 17:** Wyckoff positions of the proposed orthorhombic superstructure of the tetragonal branch with space group *C*222_1_ (No. 20) and lattice parameters 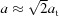, *b* ≈ *c*_t_, 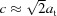

Element	Wyckoff symbol	*x*	*y*	*z*
*R*	4*a*	*x*_*R*_ ≈ ¼	0	0
*R*	4*b*	0	*y*_*R*_ ≈ ¼	¼
*T*	4*b*	*x*_*T*_ ≈ 0	*y*_*T*_ ≈ 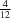	*z*_*T*_ ≈ ¼
Si	4*b*	*x*_Si,1_ ≈ 0	*y*_Si,1_ ≈ 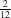	*z*_Si,1_ ≈ ¼
Si	8*c*	*x*_Si,2_ ≈ ¼	*y*_Si,2_ ≈ 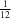	*z*_Si,2_ ≈ 0

**Table 18:** Formation energies (eV) and lattice parameters (Å) calculated with DFT Formation energies are given for *R*_2_Si_4_ and *R*_2_ *T*Si_3_ compounds, respectively (same amount of atoms within calculated range). Compounds marked with * have already been reported in the literature.

		Reported			Calculated			
Compound	Structure type	*a*	*b*	*c*	*a*	*b*	*c*	Δ*E* ^tot^
*Co series*								
La_2_CoSi_3_	Ce_2_CoSi_3_*	8.185	*a*	4.350	8.14	*a*	4.34	−4.52
Ce_2_CoSi_3_	Ce_2_CoSi_3_*	8.110	*a*	4.220	8.01	*a*	4.08	−4.61
Pr_2_CoSi_3_	Ce_2_CoSi_3_	–	–	–	8.03	*a*	4.11	−4.58
La_2_CoSi_3_	Ce_2_CoSi_3_*	8.185	*a*	4.350	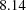	*a*	4.34	−4.52
Ce_2_CoSi_3_	Ce_2_CoSi_3_*	8.110	*a*	4.220	8.01	*a*	4.08	−4.61
Pr_2_CoSi_3_	Ce_2_CoSi_3_	–	–	–	8.03	*a*	4.11	−4.58
Nd_2_CoSi_3_	Ce_2_CoSi_3_	–	–	–	8.04	*a*	4.15	−4.37

*Rh series*								
Eu_2_RhSi_3_	Ce_2_CoSi_3_	–	–	–	8.26	*a*	4.27	−4.35
	Er_2_RhSi_3_	–	–	–	8.26	*a*	8.55	−4.34
Gd_2_RhSi_3_	Er_2_RhSi_3_*	8.112	*a*	7.976	8.21	*a*	8.02	−6.68
Tb_2_RhSi_3_	Er_2_RhSi_3_*	8.110	*a*	7.860	8.18	*a*	7.90	−5.51
Dy_2_RhSi_3_	Er_2_RhSi_3_*	8.097	*a*	7.823	8.18	*a*	7.90	−5.45
Ho_2_RhSi_3_	Er_2_RhSi_3_*	8.086	*a*	7.804	8.18	*a*	7.89	−5.31
								
*Pt series*								
Eu_2_PtSi_3_	Ce_2_CoSi_3_	–	–	–	8.27	*a*	4.34	−5.11
	Er_2_RhSi_3_	–	–	–	8.27	*a*	8.67	−5.11
Gd_2_PtSi_3_	Ce_2_CoSi_3_	–	–	–	8.17	*a*	4.14	−5.97
	Er_2_RhSi_3_*	8.139	*a*	8.303	8.17	8.17	8.28	−5.97
Tb_2_PtSi_3_	Ce_2_CoSi_3_	–	–	–	8.15	*a*	4.08	−5.92
	Er_2_RhSi_3_ ( 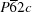 )*	8.122	*a*	8.237	8.16	*a*	8.18	−6.17
	*P*1	–	–	–	8.16	*a*	8.17	−6.18
Dy_2_PtSi_3_	Ce_2_CoSi_3_	–	–	–	8.16	*a*	4.07	−5.84
	Er_2_RhSi_3_ ( 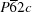 )*	–	–	–	8.22	8.23	8.33	−6.14
	*P*1	8.100	*a*	8.200	8.16	*a*	8.14	−6.14
Ho_2_PtSi_3_	Ce_2_CoSi_3_	–	–	–	8.16	*a*	4.07	−5.77
	Er_2_RhSi_3_	–	–	–	8.16	*a*	8.13	−6.04
	Er_2_RhSi_3_ ( 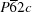 )	–	–	–	8.16	8.16	8.10	−6.04
	*P*1	–	–	–	8.16	*a*	8.11	−6.05
								
*La_2_PdSi_3_*								
La_2_PdSi_3_	Ce_2_CoSi_3_*	–	–	–	8.34	*a*	4.38	−5.54
								
*SrSi_2_ versus BaSi_2_*								
SrSi_2_	ThSi_2_*	4.438	*a*	13.830	4.46	4.46	13.82	−2.21
	AlB_2_	–	–	–	4.14	*a*	4.64	−1.90
BaSi_2_	ThSi_2_	–	–	–	4.67	4.67	14.16	−2.06
	AlB_2_	–	–	–	4.17	*a*	5.06	−2.06
								
*Sr_2_AgSi_3_ versus Ba_2_AgSi_3_*								
Sr_2_AgSi_3_	Ba_4_Li_2_Si_6_	–	–	–	8.48	14.69	18.56	−2.83
	Ca_2_AgSi_3_	–	–	–	8.48	9.28	14.67	−2.74
Ba_2_AgSi_3_	Ba_4_Li_2_Si_6_*	8.613	14.927	19.639	8.63	14.97	19.84	−2.74
	Ca_2_AgSi_3_	–	–	–	9.11	10.19	15.58	−2.36
								
*Potential tetragonal structure with ordered Si/*T* sites*								
NdSi_2_	ThSi_2_*	3.968	*a*	13.715	4.12	*a*	14.05	−3.97
	AlB_2_	–	–	–	4.08	*a*	4.13	−4.20
Nd_2_AgSi_3_	ThSi_2_*	4.175	*a*	14.310	–	–	–	–
	POTS	–	–	–	5.96	5.93	14.54	−3.72
	Ce_2_CoSi_3_	–	–	–	8.35	*a*	4.28	−3.69
Nd_2_PdSi_3_	AlB_2_*	4.103	*a*	4.204	–	–	–	–
	Ce_2_CoSi_3_	–	–	–	8.26	*a*	4.24	−5.17
Nd_2_CuSi_3_	Ce_2_CoSi_3_	–	–	–	8.06	*a*	4.26	−4.23
	Er_2_RhSi_3_ ( 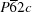 )*	8.076	*a*	8.440	8.07	*a*	8.46	−4.54
	*P*1	–	–	–	8.06	*a*	8.44	−4.14
Nd_2_NiSi_3_	Ce_2_CoSi_3_*	4.020	*a*	4.190	7.98	*a*	4.14	−6.32

**Table 19:** Space groups of the unary *R* crystals used for standardization of the formation energies

Atomic number	Element	Space group	ICSD code
14	Si	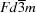 (No. 227)	51688
27	Co	*P*6_3_/*mmc* (No. 194)	184251
28	Ni	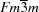 (No. 225)	646089
38	Sr	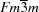 (No. 225)	652875
45	Rh	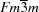 (No. 225)	171677
46	Pd	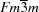 (No. 225)	76148
47	Ag	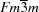 (No. 225)	181730
56	Ba	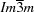 (No. 229)	108091
57	La	*P*6_3_/*mmc* (No. 194)	641382
58	Ce	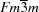 (No. 225)	620620
59	Pr	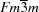 (No. 225)	649185
60	Nd	*P*6_3_/*mmc* (No. 194)	164281
63	Eu	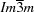 (No. 229)	604033
64	Gd	*P*6_3_/*mmc* (No. 194)	184250
65	Tb	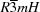 (No. 166)	652944
66	Dy	*P*6_3_/*mmc* (No. 194)	95172
67	Ho	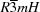 (No. 166)	639322
78	Pt	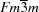 (No. 225)	649490

## Appendix A Wyckoff positions of the different superstructures

Wyckoff positions of the different superstructures are presented here in Tables 2 ▸, 3 ▸, 4 ▸, 5 ▸, 6 ▸, 7 ▸, 8 ▸, 9 ▸, 10 ▸, 11 ▸, 12 ▸, 13 ▸, 14 ▸, 15 ▸, 16 ▸ and 17 ▸.

## Appendix B Fundamentals of the DFT calculations

To calculate the formation energies with DFT, it is necessary to know the energy of the components that make up the compound. Table 19 ▸ contains a list of the underlying single-element compounds used to calculate the formation energies in Table 18 ▸.
